# Exploring the relationship between inhibitory control and coping behaviour in horses

**DOI:** 10.1038/s41598-026-48050-z

**Published:** 2026-04-18

**Authors:** Marie von der Tann, Rupert Palme, Uta König von Borstel, Désirée Brucks

**Affiliations:** 1 Animal Husbandry & Ethology, Albrecht Daniel Thaer-Institute of Agricultural and Horticultural Sciences, Faculty of Life Sciences, Humboldt-Universität zu Berlin, Berlin, Germany; 2 Department of Biological Sciences and Pathobiology, University of Veterinary Medicine Vienna, Vienna, Austria; 3 Department of Animal Breeding and Genetics, University of Giessen, Giessen, Germany

**Keywords:** Inhibitory control, Equines, Coping, Stress, Behavioural inhibition, Impulsivity, Behavioural flexibility, Neuroscience, Psychology, Psychology

## Abstract

The ability to flexibly adapt to the demands of a stressful situation is a key factor in successful coping. Appropriate coping is of critical importance in artificial environments, which may include novel and potentially stressful situations. Being able to inhibit immediate but counterproductive reactions, and thus exert inhibitory control, might allow to better adapt to the environment. Horses are required to flexibly adapt to various housing conditions and training techniques; however, not all horses successfully cope as evidenced by the prevalence of stereotypical behaviours. We tested 31 mares in an inhibitory control test battery (A-not-B detour test, delay of gratification test, reversal learning test) and assessed their behavioural and stress-physiological reaction during two husbandry conditions (social isolation and food omission). All three inhibition tests revealed behaviours indicative of inhibitory control, which could be well represented by four components resulting from a PCA (inhibition, indecisiveness, learning capacity, flexibility). The variation in coping behaviour could be captured by six components (nervousness, stress, anticipation, reactivity, oral motivation, vigilance). We found that reactivity and vigilance were positively related to inhibition. Reactivity was additionally negatively related to indecisiveness and positively related to flexibility. These findings may suggest that inhibitory control is an underlying mechanism involved in flexible responding, which may ultimately facilitate successful coping.

**Supplementary Information:**

The online version contains supplementary material available at 10.1038/s41598-026-48050-z.

## Introduction

Individuals differ in their reaction to various environmental conditions and stimuli^1^, particularly when faced with uncontrollable aversive stimuli, which are often experienced in captive conditions^2^. At one extreme, some animals react strongly to such environmental challenges and are highly agitated, while, at the other extreme, animals may remain calm and simply take notice of the stimulus. The ability to exert behavioural and physiological effort to meet the demands of a potentially stressful situation, allows individuals to adapt to various environmental conditions^3^. These response patterns often remain consistent over time and across different situations, and can be categorised as distinct coping styles or personality dimensions^3^. Proactive individuals exhibit aggression, high activity and lower HPA axis reactivity, and they are more exploratory and willing to take risks in potentially dangerous situations^4^. In contrast, reactive individuals exhibit little aggression, are less active, have a higher HPA axis reactivity, and are generally risk-averse^4^. Both types of behavioural reactions have advantages in different environmental conditions (see^5^ for a review). Proactive individuals might be better adapted to stable environmental conditions, while reactive individuals fare better in variable and unstable environments^4^. In artificial environments, which often have many novel and potentially stressful aspects, failures of coping can be exacerbated, leading to health problems^6^ and abnormal behaviours^7^. Consequently, understanding the causes and consequences of individual variation in behaviour is of particular interest for animal welfare^7^.

Behavioural flexibility—the ability that allows individuals to overcome instinctual behaviours and adapt to variation of the environment^8^—has been suggested as an underlying attribute or fundamental characteristic of coping behaviour^9^. Accordingly, successfully coping individuals are able to quickly adjust their behaviour in line with the given requirements of a situation. A range of cognitive control functions are involved in exerting behavioural flexibility. These include the ability to quickly update information, pay attention to relevant stimuli, and also employ behavioural inhibition^10^. In an early study^11^, reactive mice (*Mus musculus*) were shown to quickly adapt to changes in the light-cycle, while more proactively coping individuals needed days to adjust their activity behaviour. At the same time, reactive mice were also more attentive to novel environmental stimuli, which distracted them from completing an already learned task, while proactive individuals did not pay attention to the novel stimuli and completed the task as before^12^. Sticking with rigid and routine-like response patterns might help in situations that do not require flexible responding, however, if adjustments in behaviour are needed, reactive individuals are more successful. For example, proactively coping pigs (*Sus scrofa domesticus*) showed reduced behavioural flexibility (i.e. inhibiting previously learned response patterns) in a reversal-learning task^13^. This shows that individual responding to environmental changes, as well as the selective processing of relevant information for learning and memory, is affected by coping behaviour^14^.

One aspect of behavioural flexibility that has received considerable scientific attention due its involvement in various aspects of daily life, is inhibitory control (see^15^ for a review). Inhibitory control is the ability to inhibit certain impulsive behaviours in favour of more appropriate and advantageous behaviours in a given situation or context^16^. For example, in species with a strict dominance hierarchy, low-ranking individuals must refrain from approaching a food source directly^17^ (at least if resource competition is not extremely high^18)^. Instead, they must wait until higher-ranking individuals retreat. Ultimately, inhibitory control allows goal-directed behaviours to be enacted, even in conflicting or challenging situations, and irrelevant information to be filtered out^19^. Humans with better inhibitory control abilities are more successful in life (i.e. better health, higher success in jobs, and less prone to commit crimes^20,21^, but see^22)^ and chimpanzees (*Pan troglodytes*) that exhibited better self-control were more successful in a cognitive test battery^23^. In hamsters (*Mesocricetus auratus*)^24^, mice (e.g.^25^), rats (*Rattus norvegicus*)^26^, dogs (*Canis lupus familiaris*)^27^, and long-tailed macaques (*Macaca fascicularis*)^28^, a lack of inhibitory control has been linked to higher aggression. For chickens (*Gallus gallus domesticus*), it was found that inhibition abilities are related to affective states and fearful behaviour as measured in a cognitive judgement bias test^29^. Furthermore, stereotypical behaviour seems to be linked to diminished inhibitory control in bank voles (*Clethrionomys glareolus*)^30^ and two tit species^31^. These studies suggest that inhibitory control might be a mediating factor in coping responses. However, further studies are necessary that consider the multifaceted nature of inhibitory control (e.g.^32,33^) instead of relying on a single test for assessing inhibitory control (see^34^ for a discussion). In fact, inhibitory control is a behavioural construct that consists of at least three different domains^16^. *Self- control* is considered the choice between an immediate inferior option and a delayed preferred outcome^16^. *Motor inhibition* describes the ability to resist an immediate urge to perform a motor action and instead refrain from the action in favour of a better alternative^16^. And *cognitive inhibition* is related to learning flexibility as previously learned associations need to be quickly switched to accommodate novel circumstances; thus, requiring inhibition of previously learned associations^16^.

To further explore the relationship between inhibitory control and coping behaviour, we focused on horses (*Equus caballus*). Horses are subjected to various handling, housing, and training techniques that require coping with uncontrollable situations, which might be modulated by inhibitory control. Many horses, for example, are still kept in individual stables with limited access to outdoor space or social contact, which poses a welfare concern as they restrict the horse in expressing highly motivated behaviours (e.g.^35^). Furthermore, also from the human perspective, it is important to understand why some horses fail to cope with these conditions as the development of stereotypies^36^, aggressive behaviours or general unresponsiveness to the environment^37^ are associated with limited training success^38,39^. Horses’ reactions to stressors can be classified into distinct coping styles^39,40^ and stereotypical behaviours may be related to stress-sensitivity^41^(but see^42^), and lower behavioural flexibility^43^(but see^44^). While such differences in coping capacities have been partially attributed to personality differences and genetics^45,46^, it is not clear whether other fundamental components, such as inhibitory control, also affect horses’ coping behaviours. Considering that sport and leisure horses are required to quickly adapt to novel situations that are often in stark contrast to natural situations (e.g. loading on trailers, housing in boxes, limited access to roughage), individual differences in inhibitory control abilities might govern how quickly and how well the horses are able to adapt and cope with these conditions.

In the current study, we aimed to explore the link between inhibitory control and horses’ behavioural and physiological reactions to stressful situations. We focused on horses that were already required to actively cope with suboptimal conditions (i.e. individual stables with limited turnout) prior to the start of the study. The horses’ inhibitory control abilities were captured using a test battery consisting of three commonly used and already established tests for horses to capture different aspects of IC^15,16^, i.e. a spatial A-not-B test for testing response inhibition^47^, a delay of gratification test to test for self-control^48^, and a reversal learning test to assess cognitive inhibition^44^. Coping behaviour was observed in two different potentially stressful situations (i.e. social isolation and food omission), in which salivary cortisol concentrations were quantified. If horses’ ability to cope with stressful situations is mediated by inhibitory control, we would expect to see horses with better inhibitory control show less stress during the coping tests (i.e. less agitation and lower cortisol concentrations) compared to horses with poor inhibitory control.

## Methods

### Horses and housing

Taking the individual level as the unit of replication, we calculated that approximately 31 animals would be required, based on stable estimates of the necessary models, which should have error degrees of freedom that are at least three times as high as degrees of freedom for the fixed effects. We tested 31 female horses (age: median = 16.8 yrs.; range: 6.6–24.8 yrs.; breeds: 29 warmblood/2 thoroughbred) housed at a stud farm in Northern Germany from January - April 2024. The mares were used for breeding, but none of them were pregnant at the start of the study. Seven of the mares were inseminated at the end of the study period (4–48 days into pregnancy at last test). The horses were not ridden, and, apart from two mares exhibiting wind-sucking, none showed other stereotypical behaviours (e.g. weaving or stable-walking) at the start of the study, as described by the stable staff. During the winter months, they were kept in individual stables (3.5 × 3.5 m without window or 4 × 5 m with a front window to the aisle; height of stable walls: 1–1.6 m with metal bars up to 2.5 m) and were given access to their own paddock every other day for 4–5 h, either in the morning or afternoon. All horses from one building were led outside collectively. The horses were able to make physical contact with each other through the stable bars or directly across the paddock fences. In summer and autumn, the mares were kept permanently on pastures in same-sex groups. The horses were housed in five different buildings with 7–9 stables each. All horses were familiar with being kept in individual stables prior to the study period and were housed at the test site for at least one week before testing began (mean time at test site: median = 14.5 weeks.; range = 1-142 weeks). The horses were fed concentrate feed (ca. 1.5 kg) and hay (ca. 7 kg) twice per day (ca. 8:00 h and 17:00 h) and received haylage (ca. 2 kg) at noon (ca. 13:00 h). Ad libitum access to water was ensured via automated drinkers in all stables and on all paddocks. All stables were littered with straw and cleaned daily.

### Inhibition tests

To allow for better comparability with the literature, we selected inhibitory control tests that are established and have been conducted with horses in the past^44,47,48^ (although using modified setups). The horses were tested in three different inhibition tests in a balanced and randomised order (i.e. progressing to the next test once criteria were reached; see Table S1 for sequences of tests). To avoid potential frustration in horses waiting for their test session and to prevent social facilitation when horses observed test sessions of other horses, the daily order of testing across and within buildings was randomised. Testing started every weekday at 9:30 and was completed by 15:00, with a one-hour break after the horses were given their haylage at noon. Each horse was tested in one test per day with a maximum of two test sessions. If a horse completed one inhibition test, the next inhibition test was only started the next day. All tests were video recorded.

#### A-not-B test

In the A-not-B Test, the horses were trained to detour hurdles to reach a food reward to one side in the A-phase (right: 17 horses; left: 14 horses), and once they reliably performed the detour, the hurdles were rearranged, and the gap was shifted to the opposite side (B-trials).

**Fig. 1:**
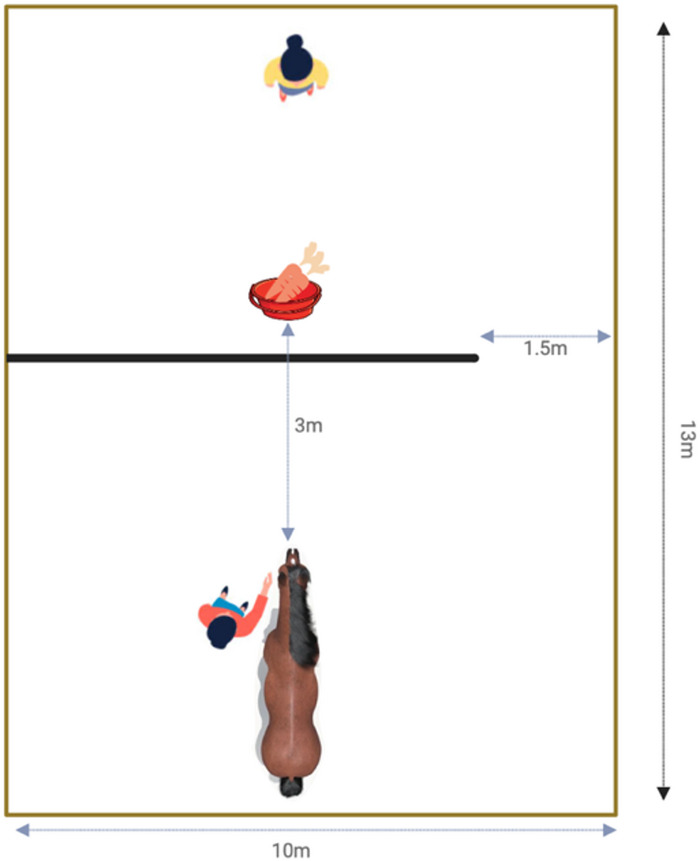
Schematic overview of A-not-B Test setup.

Following the procedure of Osthaus et al.^47^, we started with an initial exploration of the paddock and habituation to feed from the bucket. A second experimenter led the horse to the starting point in the middle of the hurdles, while the first experimenter was standing behind the hurdles with the bucket in her hands (see Supplementary Material (SM) for detailed description). The hurdles were arranged in a way that on either the left or the right side a small gap (1.5 m) was retained (Fig. 1). The side of the gap was randomly assigned for each horse (right: 17 horses; left: 14 horses). At the beginning of each trial, the second experimenter positioned the horse at the starting point, while the first experimenter visibly dropped a hand full of carrot pieces into the bucket, shook it, and placed it on the ground before leaving the paddock and turning their back to the horse and standing within 0.5 m to the fence. The second experimenter released the horse as soon as the bucket was positioned on the ground and left the paddock in a straight line towards the back of the horse. The horse had 60 s time to detour the hurdles before a trial ended. The maximum trial duration was set based on Osthaus et al.^47^, to avoid frustration and distraction. If a horse did not find the gap within this time, she was collected again by the second experimenter and led to the starting position. If a horse successfully detoured the hurdles in three consecutive trials, the A-phase was terminated and the B-phase started. Due to the decreasing motivation of horses, a maximum of 12 trials (A-phase + B-phase) were performed per session. If the horse did not reach the criterion, the session was terminated and repeated the next day. In the B-phase, the gap was moved to the opposite side. The procedure was identical to that of the A-phase. However, to fully capture the horses’ success after the switch, five B-trials were conducted, regardless of the horses’ success. No learning criterion was applied.

We analysed three variables: the accuracy of initially going to side of the gap (in A- and B-phase), the latency to cross the gap (in A- and B-phase), and the number of trials needed to reach the learning criterion in the A-phase (see Table 1 for more details).

#### Delay of gratification test

**Fig. 2:**
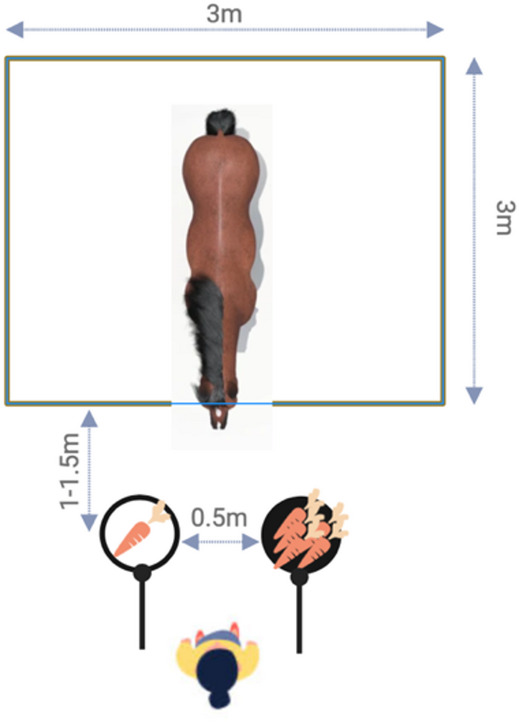
Schematic overview of the setup for the delay of gratification test.

In the Delay of Gratification Test, the horses were presented with two food options of different quantity (1 vs. 5 pieces of carrot; see Fig. 2). The higher quantity reward was only made available after a delay if the horse refrained from consuming the immediately available low-quantity reward. The test procedure was similar to Brucks et al.^48^, however the rewards were presented on plates and controls were implemented. The Delay of Gratification Test was conducted in front of the horse’s stable at a lower level to ensure maximum visual acuity^49^. We used two differently coloured plates (black and white), and each horse was assigned one colour for the high-quantity reward (HQR; 5 pieces of carrot; black: 15 horses, white: 16 horses) and one colour for the low-quantity reward (LQR; 1 piece of carrot).

The horses were habituated to feeding from the plates and were tested for their quantity discrimination capacities prior to starting the Delay of Gratification Test (see Supplementary Material for details).

In the training phase, we familiarised the horses with the test procedure by moving the LQR plate within reach 1s before moving the HQR plate. The plates remained on predetermined sides for the remaining phases (white: L (*N* = 9)/R (*N* = 7); black: L (*N* = 7)/R (*N* = 8)). The same criterion as in the quantity discrimination test was applied (10/12 HQR choices) for proceeding to the test phase (see Table S3). If a horse did not reach this criterion within 6 sessions, the test was terminated and a maximally tolerated delay of 0 s noted (*N* = 5 horses).

In the test phase, the delay between LQR and HQR was consecutively increased, from 2 s to 5 s, 10 s, 15 s, and up to 20 s, depending on the horses’ success. Based on results from a previous study^48^, we selected 20 s as the maximum delay stage, as most horses either failed before this delay stage or waited much longer. The procedure was identical to the training phase. Twelve test trials were conducted, and three control trials were randomly interspersed per session (see Table S4 for an example sequence). Three different control trials were implemented to rule out that the horses followed simple associative rules instead of paying attention to the content of the plates: (1) HQR control: HQR presented on both plates, (2) LQR control: LQR on both plates, and (3) position control: HQR plate was made accessible first; thus, reversing the normal test order. The control trials were conducted with the same delay between plates as the test trials. If a horse waited for the HQR plate in at least 4 test trials per session in two consecutive sessions (similar to^50^), she moved to the next delay stage. If a horse did not reach this criterion within six sessions, the test was terminated. A maximum of two sessions were conducted per day with a short break between sessions.

We analysed three variables: the maximally tolerated delay, the number of sessions needed to reach the criterion in the quantity discrimination test (QDT), and the duration of specific waiting behaviours (i.e. pawing and turning head away from food; see Table 1 for more details).

#### Reversal learning test

**Fig. 3:**
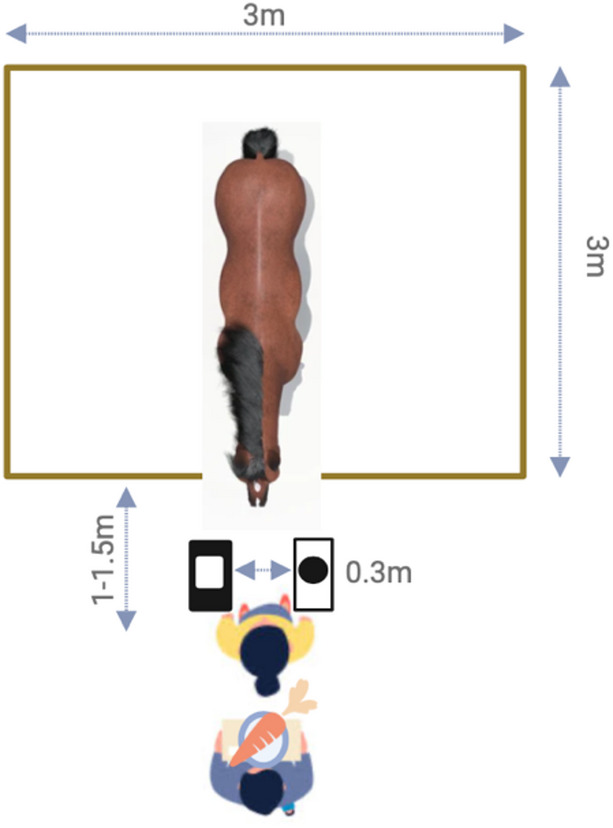
Schematic overview test procedure reversal-learning test.

In the Reversal Learning Test, the horses were trained to discriminate between two stimuli (S + and S−) and once they had reached a learning criterion, the reward contingencies were reversed (similar to^44^). Testing was conducted in front of the horse’s box. The experimenter presented two symbols (black circle (diameter: 19 cm) on white background and white square (19 × 19 cm) on black background) printed on solid A3 pages at an equal distance to the horse’s head (Fig. 3; see SM for details). Horses were randomly assigned to an S+ symbol (circle: 16 horses; square: 15 horses). A second experimenter was standing behind the first experimenter, holding a small plate (diameter: 20 cm) with a piece of carrot to reward the horse in case of a correct choice (i.e. touching symbol with nose). The food reward was not visible to the horse before making a choice.

Horses were trained to touch the S+-Symbol with their nose for receiving a food reward prior to starting the test phase (see habituation procedure in SM). The subsequent test phase consisted of two phases, the acquisition phase, in which horses initially learned to discriminate between S + and S-, and the reversal phase, in which the reward contingencies were reversed (S + now becoming S-). The test procedure was identical in both phases. The presentation of the stimuli was semi-randomised (i.e. not more than twice on same side and counterbalanced within sessions). Twelve trials were conducted per session with a maximum of two sessions per day and a break of at least 5 min in-between sessions. Once a horse made 10 S+ choices within a session, the learning criterion was accomplished (10 correct choices out of 12 trials; two-sided binomial test against chance level: *p* = 0.038; see^51^ for a review on different learning thresholds), and the reward contingencies were switched in the following reversal phase. If a horse did not reach the learning criterion within 20 sessions, the test was terminated (*N* = 1 horse). Horses needed to reach the same learning criterion in the reversal phase (10 S+ choice in one session) to successfully complete the Reversal Learning Test. If a horse did not reach the learning criterion in the reversal phase within 20 sessions, the test was terminated (*N* = 3 horses).

We analysed three variables: the number of sessions needed to reach the acquisition and reversal criterion, the number of correct choices in the 1st reversal phase, and the choice latency during correct trials across all phases (see Table 1 for details).

### Coping tests

To assess how the horses behave in a stressful situation that could resemble a standard situation in bigger equestrian facilities, we ran coping tests. Coping tests were conducted only in the morning between 7 and 9 a.m. Each coping test lasted 15 min, in which we observed the behaviour via a camera mounted on a tripod outside of the stable. Horses were tested in two different coping contexts (food omission/delayed feeding and social isolation/delayed turnout), with three repetitions each, always alternating between the two different contexts. Horses experienced only one coping test per week as the focal horse and were not tested in inhibition tests on the days on which they acted as the focal horse. We always tested two focal horses per building at the same time that both received the same treatment. Partner horses were selected based on the assigned order of coping test sequences and varied between sessions whenever possible (always a different partner per session: *N* = 19 horses; twice same partner: *N* = 12 horses; always same partner: *N* = 2 horses).

In the *food coping context*, all horses in the building, except for the two focal horses, received their normal morning feeding routine provided by the familiar stable staff (similar context as in^52^). Firstly, hay was provided to each horse by moving a portion from the hay bale to each stable individually. Then, concentrate feed was provided from a common bucket to each horse’s feeding trough. The two focal horses did not receive any food or social interactions with the stable staff during the treatment period. The feeding procedure took around 4–5 min, after which no further disturbances were happening in the building for the remaining test duration of 15 min. The focal horses were fed after the second saliva sample was taken (see below).

In the *social coping context*, all horses from one building were individually led outside on to the paddocks while the two focal horses remained in the building within visual distance of each other (similar context as in^53^). Depending on the number of horses kept in each building (one building with 6 stables, other building with 9 stables), the procedure took around 3–4 min after which no further disturbances were happening in the building. The two remaining horses were collectively led outside after the 15 min observation period had finished and the second saliva sample was taken on the paddock (see below).

#### Saliva samples

Before and after each coping test, we sampled saliva to analyse cortisol concentrations. Fifteen minutes before the start of the coping test, we collected the first saliva sample (baseline measurement). Fifteen minutes after the end of the coping test (i.e. 45 min after the first saliva sample was collected), we collected the second sample (the stress measurement). This allowed us to capture the changes in cortisol concentrations reflecting the horses’ HPA responses to the coping test, considering that it takes around 20–30 min for cortisol to reach the saliva^54^. To ensure that food residue did not interfere with the cortisol^55^, horses were not fed prior to taking the saliva samples; however, since all boxes were littered with straw, we were not able to exclude the possibility for horses to eat straw and remaining hay from the previous ration.

The saliva samples were taken using a flexible perforated PVC tube that was equipped with a synthetic swab (Salivette^®^ Cortisol; Fig. S1). Attached to the tube were two hooks and a rubber band that could be extended and pulled over the ears of the horse (similar to^56^). The tube was gently inserted in the horse’s mouth by the experimenters, and once the tube was in place, the horse was free to move for 2 min, after which the experimenter collected the tube again. The swab was pushed outside of the tube using gloves, inserted into a Salivette plastic container, and immediately transferred onto ice. Once all samples of the day had been collected, the samples were transferred to the freezer ( − 20 °C). The mouth tubes were cleaned under water and rinsed with 90% ethanol after each usage and only reused the next day. The samples remained in the freezer for a max. of 6 months before the samples were defrosted and centrifuged at 12.000U/min for 10 min at 15 °C and the extracted saliva was frozen again for the transport to the laboratory. Cortisol concentrations (in ng/ml) were determined using a cortisol enzyme immunoassay^57^ that has been validated for horses^58^.

### Ethical note

All methods were carried out in accordance with relevant guidelines and regulations (i.e.^59^). The study was approved by the Committee of Animal Use and Care of the Federal State Office for Agriculture, Food Safety, and Fisheries Mecklenburg-Vorpommern, Germany (Ref. No.: 7221.3-10099_23) to be conducted at this stud farm and was not considered to be an animal experiment within the meaning of the law, as no pain, suffering or injury was expected to be inflicted on the animals during the course of the study. The stud farm gave informed consent prior to starting data collection. All methods are reported in accordance with ARRIVE guidelines^60^.

All tests were performed in a familiar environment with other horses within a visual distance (in case of delayed turnout coping test) or with direct physical contact (all inhibition tests and delayed feeding coping test). From autumn to early spring (when the data for this study was collected), the horses were given the opportunity to exercise on paddocks every other day. They were fed three times a day and there was remaining hay and straw in their stables throughout the day. Social contact was possible via the fences of the paddocks and through the bars of the stables. During summer months, the horses were kept on pastures in groups and did not receive additional food. All horses were accustomed to being handled, led by the halter and rope and were used to interacting with different people. The coping tests simulated situations potentially occurring during daily husbandry procedures (i.e. delayed feeding during morning feeding routine and delayed turnout during usual morning turnout time). Two horses were always tested in the building at a time to prevent complete isolation for the focal horse, with the aim of keeping stress responses during the coping tests (i.e. delayed turnout), at an acceptable level. Saliva samples were collected using an adapted procedure (see above) to avoid unnecessary restraint. If a horse showed signs of stress during the inhibition tests (i.e. neighing, excessive locomotion, alert posture), the test was terminated and repeated at a later time (*N* = 9 sessions).

### Analyses

All coping and inhibition tests were video recorded and analysed using Solomon Coder (^©^2017 by András Péter). We coded the frequency and duration of various behaviours using continuous sampling (see Tables 1 and 2). A second coder coded 20% of each test to assess inter-rater reliability. Intra-class correlation coefficients (ICC; two-way, agreement) were calculated for each variable using R (Version 4.2.2.;^61^) and the *irr* package^62^. The variables showed moderate to excellent reliability^63^, with ICCs ranging from 0.603 to 0.999 (see Table S5).

**Table 1:** Overview of all variables that were coded per inhibitory control test along with the descriptive statistics of variables that were used for the PCA.

Variable	Definition	Median and Range
**A-not-B Test**		
Accuracy	1st step towards either side of hurdles (per trial)	A-phase: 3 correct (range: 0–3) B-phase: 2 correct (range: 0–5)
Latency gap	Latency to cross gap with hoof (per trial)	A3: 8.4 s (range: 2.8–58) B1: 26 s (range: 5.8–60)
Trials to A-criterion	Total number of trials needed to reach learning criterion	6 trials (range: 3–25)
**Delay of Gratification Test**		
Choice	Eating from either plate (per trial)	Choice for 2nd plate per session (range: 0–12)
Waiting behaviour	Proportion of test duration exhibiting behaviours (per session): *Pawing* (lifting and repeated forward motion with one hoof) or *Turn away* (head turned at > 45° angle from experimenter)	Pawing: 0.0s (range: 0-16.4) Turn away: 0.0s (range: 0-22.6)
Maximum delay	Highest delay stage that was successfully mastered	2s delay (range: 0–20)
Sessions QDT	Sessions needed to reach quantity discrimination test criterion	4 sessions (range: 1–10 sessions)
**Reversal-Learning Test**		
Choice	Touching either symbol	7 correct choices (range: 0–12)
Latency choice	Latency to select either symbol	3.3s (range: 0.4–20)
Sessions to acquisition criterion	Total number of sessions needed to reach learning criterion	5 sessions (range: 1–20)
Sessions to reversal criterion	Total number of sessions needed to reach reversal criterion	7.5 sessions (range: 2–20)

**Table 2:** Ethogram of the coping tests including type of variable (based on^53,64,65^).

Variable	Definition
**Activity**	
Locomotion (duration)	Horse makes more than one step
Feed (duration)	Head is lowered to the ground and remains lowered for at least 5 s or straw is visible inside of mouth when head is raised
Alert (duration)	Head is raised higher than withers (nostrils higher than withers). Horse is standing still, while head might be scanning laterally without lowering it. Eye white can be visible.
**Vocalisations**	
Neigh (duration)	High-frequency whinny sound
Nicker (frequency)	Low-frequency rumbling sound
Snort (frequency)	Pulsed sound produced by nostrils when exhaling air
**Head movements**	
Shake (frequency)	Shaking head or whole body in a rotational manner along the body axis. Each single shaking motion is counted.
Check trough (frequency)	Lowering head into feed trough
Repetitive movements (frequency)	Repetitive head movements, including head toss (rotational movement), nod (vertical movement), and horizontal movement. New bout is counted if motionless for at least 10 s.
**Oral behaviours**	
Yawn (frequency)	Mouth wide open with deep inhalation and exhalation of air
Sham chew (frequency)	Movement of jaws and/or extension of tongue without previous feeding event
Bar-bite (frequency)	Opening mouth and closing incisors around a metal bar, movement while keeping incisors shut might follow (teeth scraping)
Repetitive oral (binary)	Repetitive oral behaviours without a clear function, including lipsmack (lips opened and closed in vertical manner), tongue-roll (mouth open with tongue visibly extended and retreated while biting on it), crib-bite/wind-suck (audible ingestion of air while placing incisors on feed trough or without contact to an object), manipulate box (licking walls or bars). These behaviours were coded as binary (observed/not observed) and summed up per session.
Groom (frequency)	Oral manipulation of body parts (e.g. nibbing, biting, licking)
**Other**	
Defecate (frequency)	Raising tail and dropping faeces (urination not counted)
Aggression (frequency)	Kicking (rapid extension of one hind leg to kick against box walls) and aggressive threats (rapid approach to neighbouring horse with ears pinned, mouth might be opened to bite)
Rear (frequency)	Rising on hind legs or attempts to rise with front feet leaving the ground
Paw (duration)	Lifting one front leg and repeatedly moving it forward and back again. Hoof can touch ground and produce scratching sound

### Statistical analyses

All analyses were performed in R (Version 4.2.2.;^61^) using the packages lme4^66^ for running all linear models and DHARMa^67^ for assessing normality and homogeneity of residuals.

Firstly, we assessed whether the inhibition tests indeed captured impulsive behaviour as anticipated. We tested whether the horses’ performance in each inhibition test differed between test phases (A-not-B Test and Reversal Learning Test) and delay stages (Delay of Gratification test) by running separate models (see Supplementary Materials for details).

Secondly, to summarise co-varying variables within and across tests, we ran a principal component analysis (PCA) using the *princomp*-function based on the correlation matrix. We included three variables from each test that captured inhibition but also learning capacity (see Table 3). To get a univariate distribution of the variables that is as normal as possible, all variables were transformed prior to the PCA (i.e. asin(sqrt), sqrt, and log; see Table 3). Missing values due to horses not finishing single tests, were imputed and the resulting PCAs visually compared (see SM and Table S7). Components with an Eigenvalue > 1 were retained and individual component scores were extracted for each horse.

**Table 3:** Overview of variables entered into inhibition PCA.

Test	Variable (transformation)	Description	Interpretation
AB	Ratio latency (log)	$\:\frac{Latency\:to\:cross\:gap\:in\:B1}{Latency\:to\:cross\:gap\:in\:A3}$	Low = high IC
	Ratio accuracy (asin)	$\:\frac{Proportion\:accuracy\:B\:trials}{Proportion\:accuracy\:A+B\:trials}$	High = high IC
	Trials to criterion (sqrt)	Number of trials to reach A-criterion	Low = quick spatial learning
DG	Maximum delay (sqrt)	Highest successful delay stage	High = high IC
	Mean waiting behaviours (sqrt)	Mean duration of pawing and turning head away while waiting across all sessions	High = better coping^1^
	Session QDT (sqrt)	Number of sessions to reach criterion in quantity discrimination test	Low = quick discrimination
RL	Success in 1st reversal (sqrt)	Number of correct trials in 1st reversal session	High = high IC
	Ratio sessions (log)	$\:\frac{\#\:session\:reversal\:phase}{\#\:session\:acquisition\:phase}$	Low = high IC
	Mean latency correct choices (log)	Mean latency to make a choice in correct trials across all sessions and phases	Low = high IC

IC = inhibitory control; AB = A-not-B test; B1 = 1st trial in B-phase; A3 = 3rd trial in A-phase; DG = Delay of Gratification Test; RL = Reversal-Learning Test; QDT = quantity discrimination test.

^1^interpretation based on positive relationship between waiting behaviours and waiting success (see ref^48^ and Fig. S5a).

Before entering the cortisol measurements into the Coping-PCA, we analysed whether concentrations differed between contexts and time points by running a linear mixed model (LMM;^68^) with log-transformed cortisol concentration as response variable and session (1–3; z-transformed), context (factor: food, social), time point (factor: before, after test), as well as an interaction between context x session, and context x time point as predictors. Horse ID nested in date was set as random effect with session, context and time point as random slopes.

For the Coping-PCA, we entered 18 behavioural (see Table 2) and two cortisol variables (see below). We had to exclude sham-chewing as this could not be coded reliably (i.e. less visible in stables with higher walls). All behavioural variables were divided by the total observation time and transformed (i.e. sqrt for oral behaviours, asin(sqrt) for all other variables) before entering them into the PCA. For the cortisol measurements, we log-transformed both measures and used the ratio between cortisol level after the coping test and the respective baseline measurement taken before the test. In instances where cortisol measurements could not be obtained (due to an insufficient volume of sampled saliva), the median values from all baseline measurements of the horse (*N* = 4 samples) or the median of the horse’s cortisol measurement in the respective context (food or social) were used to impute missing measurements (*N* = 3 samples). The PCA based on the original data set and the data set with imputed values was compared using a visualization of the strength of the factor loadings (see Table S8). Components with an Eigenvalue > 1 were retained and individual component scores were extracted for each horse. Furthermore, we assessed the temporal repeatability of the components across test sessions by calculating R^2^ (individual variance/ individual variance + residual variance;^69^).

Finally, to assess whether the horses’ behaviour captured in the coping tests could be predicted by the inhibitory control components (IC-components), we ran six separate linear mixed models, one model for each of the coping test components. We set the individual scores per coping component as the response variables. Since the age of the horses showed a skewed distribution, causing problems in the assumptions of the mixed models (i.e. they lead to highly non-linear effects), we categorised the variable into four groups: young (7–11 yrs.; *N* = 5 horses), middle (12–15 yrs.; *N* = 7), old (16–19 yrs.; *N* = 14), and senior (20–25 yrs.; *N* = 5). Age (factor: young, middle, old, senior), session (z-transformed to a mean of zero and a standard deviation of one), context (factor: food, social), and the four components derived from the IC test battery (*Inhibition*, *Indecisiveness*, *Learning capacity*, *Flexibility*), as well a two-way interaction between context and IC-components and session, were entered as predictors. We used sum contrasts for the categorical variables ‘condition’ and ‘age class’. Horse identity, building, and partner horses tested in the same session, were included as random effects, with a session x context interaction as random slope effects for the horse identity. All models were checked for collinearity (all variance inflation factors < 1.11), and graphically for normality and homogeneity of residuals. No deviations from statistical assumptions were visible in the final models. These LMMs were based on 186 coping tests of 31 horses tested in five different buildings.

## Results

### Inhibitory control tests

In all three IC tests, we found behavioural patterns that suggest that the horses needed to inhibit certain responses to the stimuli presented and instead perform other behaviours to succeed. In the AB test, we found that horses took longer to locate the gap (Proportional Hazards Model (trial A3 vs. B1): 0.777 ± 0.262, z = 2.970, *p* = 0.003; see Fig. S2a) and more frequently approached the previously open side incorrectly (GLMM (trial A3 vs. B1): − 1.599 ± 0.499, z = − 3.607, *p* = 0.002; see Fig. S3a), once the sides were switched (see Supplementary Materials for detailed results). In the DG test, horses waited less often for the delayed reward with increasing delays (GLMM: − 2.630 ± 0.630, z = − 4.173, *p* < 0.001; see Fig. S4b), and we found that horses that exhibited specific waiting behaviours (i.e. pawing, turning away from rewards) waited more often for the delayed reward (GLMM: 0.508 ± 0.055, z = 9.265, *p* < 0.001; see Fig. S5a and Supplementary Materials for detailed results). Furthermore, in the RL test, we observed that the horses were less successful once the reward contingencies were reversed (GLMM: − 0.386 ± 0.067, z = − 5.783, *p* < 0.001; see Fig. S7); however, the reversal did not affect the horses’ latency to make correct or incorrect choices (LMM: 0.008 ± 0.047, t = 0.164, *p* = 0.873, see Fig. S8).

The PCA of the inhibition variables revealed a four-component structure, which cumulatively explained 77.54% of the observed variation (see Table 4). We observed positive loadings on the first component by the two variables derived from the DG test (max. delay (= high delay tolerated) and waiting behaviours (= high occurrence of pawing and turning away from reward)) and a negative loading of ratio latency in the AB test (= fast re-learning/no perseveration errors). We named the first component ‘*Inhibition’* as these variables seem to be related to good inhibitory control (i.e. inhibition of immediate reaction and instead waiting for better reward in DG^16^ and inhibition of perseverative responding in AB task^47^).

The second component had a positive loading of latency correct choices in the RL test (= slow choices), and sessions in quantity discrimination test of the DG test (= more sessions to discriminate between quantities), as well as a negative loading of the ratio accuracy in the AB test (= low accuracy in both phases). Since these variables capture aspects of a speed-accuracy trade-off (i.e. slow reactions as well as low success in tasks that rely on learning (i.e. accuracy in AB test and quantity discrimination in DG test)), we named this component ‘*Indecisiveness’*.

A positive loading of success in the first reversal in the RL test (= more correct choices in 1st reversal session) and trials to criterion in the AB test (= more trials needed to learn pre-test criterion) were observed for the third component. These variables might be related to the horses’ learning capacity (i.e. re-learning reward contingencies in RL test, learning associations in AB test) and we named this component ‘*Learning capacity’*.

And finally, on the fourth component, we found a negative loading of the ratio of sessions in the RL test (= less sessions needed to re-learn contingencies), and a negative loading of trials to criterion in the AB test (= less trials needed to reach criterion). We named this component ‘*Flexibility’*.

**Table 4:** Loadings, eigenvalues, and explained variance of components derived from the inhibitory control tests (see Table 3 for description of variables).

	Inhibition	Indecisiveness	Learning capacity	Flexibility
DG max. delay	0.536	0.218	0.107	0.010
DG waiting behaviour	**0.528**	0.191	0.198	0.248
AB ratio latency	** − 0.474**	0.222	0.042	− 0.095
AB ratio accuracy	0.259	** − 0.532**	− 0.127	− 0.116
RL choice latency	0.107	**0.530**	− 0.226	− 0.082
DG learning criterion	0.048	**0.442**	0.198	0.115
RL success reversal	0.076	− 0.282	**0.707**	0.066
AB trials criterion	− 0.108	0.161	**0.513**	** − 0.660**
RL ratio sessions	0.332	− 0.041	− 0.281	** − 0.675**
**Eigenvalue**	2.45	2.17	1.30	1.07
**Variance**	27.17	24.06	14.44	11.87
Loadings > 0.35 are highlighted in bold as they were used for interpretation.				
AB = A-not-B Test; DG = Delay of Gratification Test; RL = Reversal Learning Test.				

### Coping tests

The duration of the stress-inducing procedure differed between the two coping contexts. It took longer to feed the other horses in the building (delayed feeding; median = 268.4 s; range = 190–496 s) than to lead the other horses outside (delayed turnout; median = 210.1 s; range: 108–345 s). The baseline cortisol concentrations did not differ between delayed feeding and delayed turnout (LMM: 0.008 ± 0.031, t = 0.293, *p* = 0.769), but the post-test samples revealed higher concentrations following the delayed turnout compared to the delayed feeding (LMM: 0.206 ± 0.031, t = 6.728, *p* < 0.001; see Fig. S9).

The coping PCA revealed six components with an explained cumulative variance of 66.93% (Table 5). We named the first component *Nervousness*, as behaviours related to increased arousal and agitation loaded positively on this component. The second component contained positive loadings of agonistic behaviours (i.e. aggression and bar-biting), and an increased baseline cortisol concentration; consequently, we named this component *Stress.* The third component was named *Anticipation* since food-related vocalisations loaded positively (i.e. nicker), while agonistic behaviours loaded negatively. Baseline cortisol and self-directed behaviour (grooming) loaded negatively on the fourth component, while cortisol difference loaded positively; accordingly, we called this component *Reactivity*. The fifth component was named *Oral motivation* because it contained positive loadings of behaviours related to mouth movements in a food context (i.e. feeding and repetitive oral behaviours (tongue-rolling, lip-smacking, crib-biting, and licking)), while having negative loadings of behaviours indicative of arousal (i.e. pawing). And finally, the sixth component had positive loadings of behaviours related to evaluating the environment (i.e. alert, check trough, nicker), we named the component *Vigilance*.

The temporal repeatability (individual variance/ (individual variance + residual variance)) of these coping components, measured across a period of six to seven weeks, ranged from low (*Reactivity*: R^2^ = 0.22, *Anticipation*: R^2^ = 0.24) to moderate (*Nervousness*: R^2^ = 0.43, *Vigilance*: R^2^ = 0.40) and high (*Stress*: R^2^ = 0.57, *Oral motivation*: R^2^ = 0.54).

**Table 5:** Loadings, eigenvalues and explained variance of the PCA based on the coping test.

	Nervousness	Stress	Anticipation	Reactivity	Oral motivation	Vigilance
Neigh	**0.369**	− 0.184	0.051	− 0.132	0.058	− 0.073
Locomotion	**0.366**	0.052	0.184	− 0.016	0.117	0.006
Defecate	**0.353**	− 0.131	0.047	0.022	0.109	− 0.011
Shake (body + head)	0.304	0.239	− 0.068	− 0.054	0.230	0.016
Snort	0.296	0.254	0.145	0.025	0.026	− 0.239
Rear	0.294	0.241	0.144	0.247	0.043	0.029
Head movements	0.288	0.199	− 0.168	− 0.025	0.100	0.250
Agonistic	0.018	**0.385**	** − 0.426**	0.068	0.032	0.333
Baseline cortisol	− 0.033	**0.383**	0.119	** − 0.546**	0.093	− 0.150
Bar bite	− 0.019	**0.369**	− 0.330	0.139	− 0.341	0.077
Nicker	− 0.027	0.034	**0.507**	− 0.155	− 0.017	**0.445**
Cortisol difference	0.230	− 0.213	− 0.118	**0.482**	− 0.185	− 0.166
Groom	− 0.096	− 0.081	− 0.349	** − 0.352**	0.083	− 0.024
Paw	0.036	0.235	0.261	− 0.100	** − 0.605**	− 0.307
Feed	− 0.261	0.008	0.099	0.176	**0.476**	− 0.143
Repetitive oral	− 0.111	0.309	0.126	0.286	**0.355**	− 0.128
Check trough	− 0.209	0.131	0.306	0.258	− 0.124	**0.453**
Alert	0.258	− 0.273	− 0.046	− 0.154	− 0.063	**0.415**
**Eigenvalue**	4.98	2.37	1.51	1.21	1.07	1.00
**Variance**	27.17	13.14	8.39	6.70	5.99	5.54
Loadings > 0.35 are highlighted in bold as they were used for interpretation.						

### Link between coping behaviour and impulsivity

For the *Nervousness* component, we found a statistically significant effect of context: horses scored lower on the *Nervousness* component in the food context compared to the social context (LMM:  − 1.862 ± 0.124, t =  − 15.0.35, *p* < 0.001; see Fig. S10b and Table 6). The *Stress* component revealed a trend for age class, with the youngest age class scoring lower than the other classes (LMM:  − 1.102 ± 0.334, t =  − 3.298, *p* = 0.055; see Fig. S11a and Table 6). Scores on the *Anticipation* component tended to decrease across sessions (LMM:  − 0.137 ± 0.076, t =  − 1.814, *p* = 0.074; see Fig. S12c; Table 6).

**Table 6:** Test statistics of likelihood-ratio tests of all main effects for the coping components (see Fig. S10-S15 for model estimates and confidence intervals of non-significant effects).

Main effects	Response variables					
	Nervousness	Stress	Anticipation	Reactivity	Oral motivation	Vigilance
Age class	*χ*^2^_3_ = 0.726, *p* = 0.867	*χ*^2^_3_ = 7.613, p = ***0.055***	*χ*^2^_3_ = 3.177, *p* = 0.365	*χ*^2^_3_ = 8.425, p = **0.038**	*χ*^2^_3_ = 2.039, *p* = 0.564	*χ*^2^_3_ = 0.077, *p* = 0.995
Context	*χ*^2^_1_ = 64.846, p **< 0.001**	*χ*^2^_1_ = 0.931, *p* = 0.335	*χ*^2^_1_ = 0.488, *p* = 0.485	*χ*^2^_1_ = 5.094, p = **0.024**	*χ*^2^_1_ = 2.345, *p* = 0.126	*χ*^2^_1_ = 1.072, *p* = 0.301
Session	*χ*^2^_1_ = 0.081, *p* = 0.776	*χ*^2^_1_ = 0.173, *p* = 0.678	*χ*^2^_1_ = 3.195, p = ***0.074***	*χ*^2^_1_ = 0.018, *p* = 0.894	*χ*^2^_1_ = 2.283, *p* = 0.131	*χ*^2^_1_ = 4.531, p = **0.033**
Inhibition	*χ*^2^_1_ = 0.024, *p* = 0.876	*χ*^2^_1_ = 0.503, *p* = 0.478	*χ*^2^_1_ = 1.476, *p* = 0.224	*χ*^2^_1_ = 3.113, p = ***0.078***	*χ*^2^_1_ = 0.001, *p* = 0.997	*χ*^2^_1_ = 0.087, *p* = 0.769
Indecisiveness	*χ*^2^_1_ = 0.994, *p* = 0.319	*χ*^2^_1_ = 0.304, *p* = 0.582	*χ*^2^_1_ = 0.122, *p* = 0.727	*χ*^2^_1_ = 5.507, p = **0.019**	*χ*^2^_1_ = 0.058, *p* = 0.771	*χ*^2^_1_ = 0.083, *p* = 0.363
Learning capacity	*χ*^2^_1_ = 0.007, *p* = 0.935	*χ*^2^_1_ = 0.403, *p* = 0.511	*χ*^2^_1_ = 0.004, *p* = 0.949	*χ*^2^_1_ = 0.170, *p* = 0.680	*χ*^2^_1_ = 1.593, *p* = 0.207	*χ*^2^_1_ = 0.061, *p* = 0.805
Flexibility	*χ*^2^_1_ = 0.119, *p* = 0.730	*χ*^2^_1_ = 0.031, *p* = 0.861	*χ*^2^_1_ = 1.449, *p* = 0.229	*χ*^2^_1_ = 2.884, *p* = 0.089	*χ*^2^_1_ = 0.183, *p* = 0.669	*χ*^2^_1_ = 0.731, *p* = 0.393
Context x Inhibition	*χ*^2^_1_ = 0.077, *p* = 0.782	*χ*^2^_1_ = 0.017, *p* = 0.898	*χ*^2^_1_ = 0.278, *p* = 0.599	*χ*^2^_1_ = 0.053, *p* = 0.818	*χ*^2^_1_ = 0.447, *p* = 0.504	*χ*^2^_1_ = 3.075, p = ***0.079***
Context x Indecisiveness	*χ*^2^_1_ = 1.522, *p* = 0.217	*χ*^2^_1_ = 0.047, *p* = 0.828	*χ*^2^_1_ = 0.015, *p* = 0.904	*χ*^2^_1_ = 4.331, *p* = 0.511	*χ*^2^_1_ = 0.026, *p* = 0.872	*χ*^2^_1_ = 0.006, *p* = 0.936
Context x Learning capacity	*χ*^2^_1_ = 0.612, *p* = 0.434	*χ*^2^_1_ = 0.134, *p* = 0.714	*χ*^2^_1_ = 1.804, *p* = 0.179	*χ*^2^_1_ = 1.195, *p* = 0.274	*χ*^2^_1_ = 1.287, *p* = 0.257	*χ*^2^_1_ = 0.588, *p* = 0.443
Context x Flexibility	*χ*^2^_1_ = 0.551, *p* = 0.458	*χ*^2^_1_ = 0.648, *p* = 0.421	*χ*^2^_1_ = 1.856, *p* = 0.173	*χ*^2^_1_ = 4.261, p = **0.039**	*χ*^2^_1_ = 0.185, *p* = 0.669	*χ*^2^_1_ = 1.149, *p* = 0.284
Context x Session	*χ*^2^_1_ = 0.953, *p* = 0.329	*χ*^2^_1_ = 1.212, *p* = 0.271	*χ*^2^_1_ = 0.212, *p* = 0.645	*χ*^2^_1_ = 0.535, *p* = 0.464	*χ*^2^_1_ = 3.003, *p* = 0.083	*χ*^2^_1_ = 2.264, *p* = 0.132
Bold numbers indicate *P*-values < 0.05; bold and italic numbers indicate *P*-values < 0.08.						

**Fig. 4:**
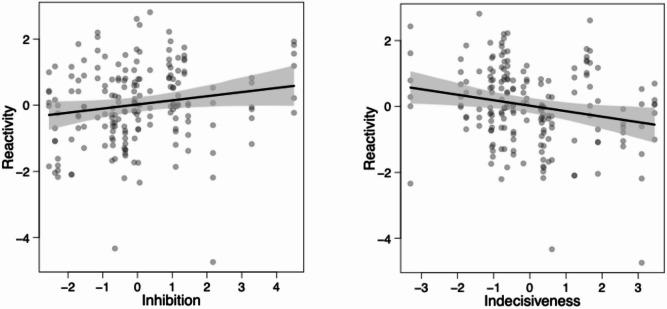
Coping component *Reactivity* as a function of the IC component (**a**) *Inhibition* and (**b**) *Indecisiveness.* The solid lines depict the fitted model and the dotted lines the respective 95% confidence intervals.

**Fig. 5:**
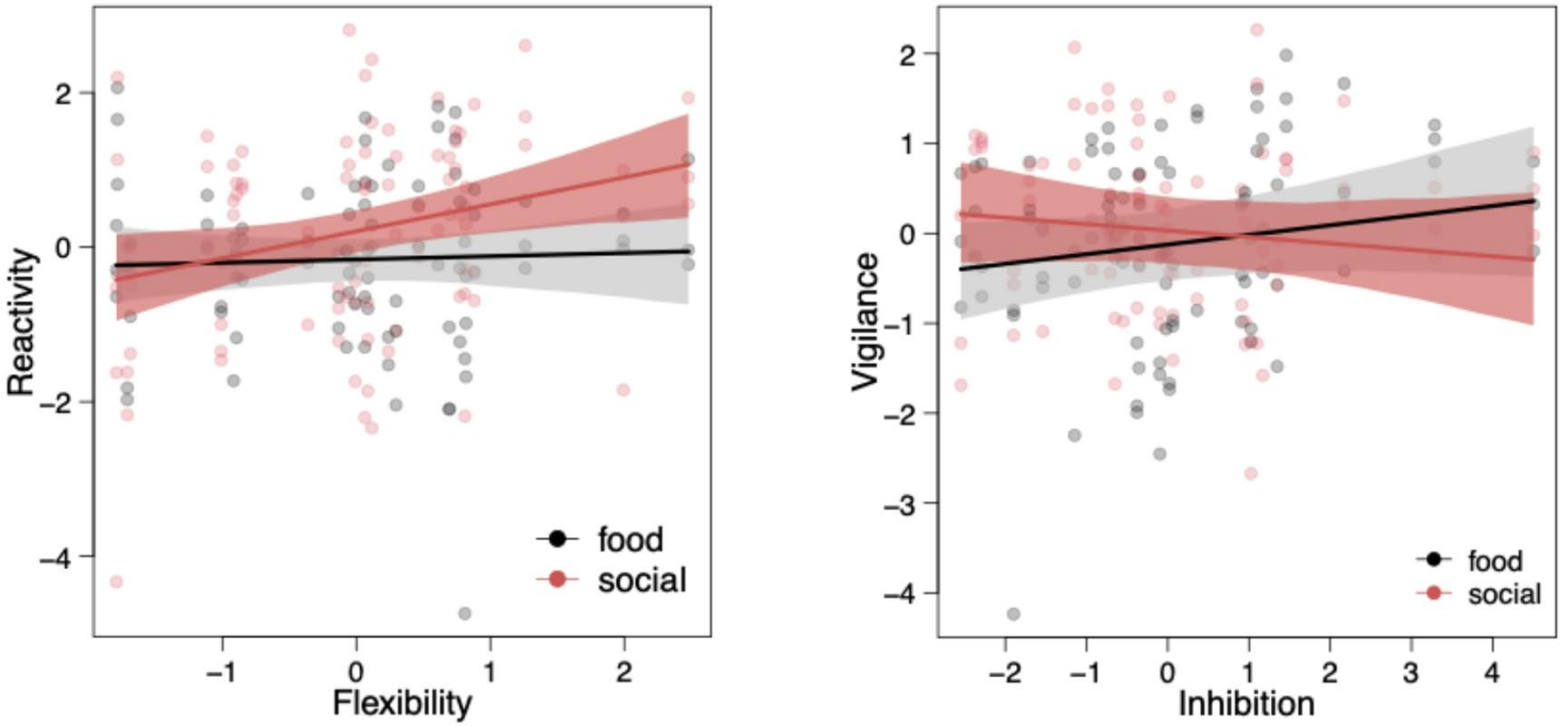
Coping Components (**a**) *Reactivity* as a function of the IC component *Flexibility* and (**b**) *Vigilance* as a function of the IC component *Inhibition*. The solid line depicts the fitted model for the food (black) and social condition (red) and the dotted lines the respective 95% confidence intervals.

The *Reactivity* component was related to age class with younger horses being less reactive than older horses (LMM:  − 0.333 ± 0.215, t =  − 1.554, *p* = 0.038; see Fig. S13a). Furthermore, horses scored higher on the *Reactivity* component in the social context compared to the food context (LMM:  − 0.182 ± 0.078, t =  − 2.345, *p* = 0.024; see Fig. S13b). *Reactivity* was also positively related to the *Inhibition* component (LMM: 0.125 ± 0.065, t = 1.931, *p* = 0.078; see Fig. 4a) and negatively related to the *Indecisiveness* component (LMM:  − 0.166 ± 0.065, t =  − 2.560, *p* = 0.019; see Fig. 4b; Table 6). Furthermore, horses scoring high on the *Reactivity* component in the social context also scored high on the *Flexibility* component (LMM:  − 0.153 ± 0.072, t =  − 2.133, *p* = 0.039; see Fig. 5a; Table 6).

And finally, for the *Vigilance* component, we observed a statistically significant effect of session and a trend for the *Inhibition* component (see Table 6). Horses scored lower on the *Vigilance* component across sessions (LMM: − 0.116 ± 0.053, t = − 2.202, *p* = 0.033; see Fig. S15c). And horses that were more vigilant in the food coping context also showed better inhibition in the IC battery (LMM: 0.089 ± 0.047, t = 1.902, *p* = 0.079; see Fig. 5b).

## Discussion

In this study, we aimed to assess horses’ inhibition capacities and investigate whether these capacities are linked to coping behaviour in two stressful situations. We found that the inhibitory control test battery elicited behavioural reactions reminiscent of inhibitory control. These measurements may capture multiple aspects of the inhibitory control tests and loaded on four components (i.e. *Inhibition*, *Indecisiveness*, *Learning capacity*, and *Flexibility*). The horses’ behaviour and physiological reaction in the coping tests could be described based on six components (i.e. *Nervousness*, *Stress*, *Anticipation*, *Reactivity*, *Oral motivation*, and *Vigilance*), and these components were partially linked to the responses in the inhibitory control tests.

### Content validity of inhibitory control tests

We were able to ensure the content validity of our inhibition measurements as the horses’ behaviour in each test was in line with the predictions (i.e. more mistakes with increasing inhibition demands). Horses committed perseveration errors in the A-not-B Test, as they needed longer and were less accurate in finding the gap once sides were switched (similar to^47,70^). Their success in waiting for the higher quantity reward decreased with increasing delay duration in the delay of gratification test, while waiting behaviours were positively associated with waiting success (similar to^48^). Also, in the reversal learning test, we observed that the horses had problems inhibiting previously learned responses when reward contingencies were reversed and exhibited a clear drop in performance in the first reversal session (similar to^44^).

Similar to other studies assessing convergent validity across inhibitory control tests^32,33,71–73^, the variables obtained from the three inhibitory control tests did not load on a single component but rather on four components, which captured a high proportion of variation (77%). We deliberately decided to include not only variables that putatively measure inhibitory control, but also variables related to learning capacity, to fully capture individual performances in the tests. In fact, the often-used main measures for inhibitory control from the three tests (i.e. AB: accuracy, DG: max. delay, RL: reversal success) loaded on separate components, whereas measures of learning performance and response speed were more consistent across tests. This separation of accuracy and response time from other inhibitory control measures, warrants further investigation in relation to the speed-accuracy trade-off theory^14^, which proposes that high accuracy often is associated with slower reaction times. Recent studies have, however, shown that not every aspect of inhibitory control seems to underlie the speed-accuracy trade-off (e.g.^74,75^). Our results indicate that inhibitory control is a multifaceted construct in horses, with each test capturing different aspects of inhibition but also general individual behavioural patterns (i.e. food motivation, decision speed, problem-solving capacity). Since we did not repeat the inhibitory control test battery, we cannot assess the temporal stability of these measurements derived from the IC tasks. However, given that cognitive processes, such as IC, can be flexibly adapted in response to variable environments (e.g. resource availability, social context), it might be promising to focus on the flexibility of IC in different conditions to gain insights into the relationship between IC, personality and cognitive styles^14^.

### Assessment of coping behaviour

The horses’ behaviour in the coping tests could be best explained by a five-component structure, which we named *Nervousness*, *Stress*, *Anticipation*, *Reactivity*, *Oral motivation*, and *Vigilance*. We observed great individual variation on all components and together with the fact that we did only observe the horses’ behaviour in two contexts, we refrain from assigning labels of coping styles to the horses. Scores on these components showed low to moderate repeatability. The social coping context (delayed turnout) elicited stronger behavioural reactions (i.e. for *Nervousness*, *Reactivity*, and *Vigilance* components), which likely captures horses’ high motivation for group cohesion, social contact and time outdoors. Interestingly, age explained variation of only two coping components. Horses belonging to the youngest age class (7–12 yrs.) scored lower on the *Stress* and *Reactivity* components compared to horses of the other age classes. This result is interesting, as older horses usually show weaker reactions to stressful situations^76^. However, it should be noted that age was negatively correlated with the time spent at the research site (Spearman: rs = -0.43, *p* < 0.001). Accordingly, these two variables are conflated. The younger horses’ habituation to the daily procedures and housing conditions due to a longer exposure might govern this age effect. Generally, similar components have emerged in other studies that assessed personality in horses (see^39^ for a review), however, it needs to be noted that we did not intend to measure personality in this study but rather focused on the behavioural reaction to two husbandry contexts. Nonetheless, further research is required to replicate the structure of the component loadings and confirm whether the coping tests were measuring reliable and usable behavioural constructs.

### Stress reactivity and food-related attentiveness are related to inhibition

The coping components *Reactivity* and *Vigilance* were related to the inhibitory control component *Inhibition*. Horses that scored higher on the *Reactivity* component tended to also score higher on the *Inhibition* component; thus, tolerating higher delay times in the DG test and showing little perseveration in the AB test. Our *Reactivity* component included negative loadings of the baseline cortisol concentration and grooming, as well as the increase in cortisol during the coping test; thus, likely capturing a general sensitivity to stress. Accordingly, one might argue that horses that can better modulate their physiological stress response (i.e. those that are less aroused by the current housing conditions and have a more attenuated response to acute stressors) exhibit better inhibitory control. In humans, cortisol reactivity seems to be linked to impulsive behaviours (e.g.^77^) and in squirrel monkeys (*Saimiri sciureus*), long-term treatment with cortisol resulted in lower response inhibition^78^. This suggests that arousal but also general sensitivity to stressors can affect inhibitory control.

Furthermore, for the *Vigilance* component, we found a stronger effect in the food context compared to the social context, which indicates that food-related cues were modulating this relationship. Horses that were more vigilant in the food context scored higher on the *Inhibition* component. The *Vigilance* component (incl. high alertness and nicker vocalisations) might capture attentiveness to the environment, in this case the arrival of food. Consequently, more attentive horses were quicker to solve the A-not-B test (as a putative measure for response inhibition) and tolerated higher delays. More attentive horses might be able to better grasp the task contingencies and thus to respond more flexible and to maintain the decision for longer. The role of food-motivation has been suggested to affect participation in cognitive tests in general, with more food motivated individuals participating quicker and more often^79^. At the same time, food motivation has been directly linked to impulsive behaviours, such as food addiction-like behaviours (e.g. in rats^80^) and perseverative responding in motor inhibition tests^81,82^, due to an inability to redirect the attention away from the food stimulus. Interestingly, though, the only other component that appears to be broadly related to the feeding period in terms of food motivation or emotions associated with the anticipation of food^83–85^ (*Oral motivation* with loadings of feeding, pawing, and repetitive oral behaviours), did not show any relationships with the inhibition components. Potentially, we have reduced the variation in food motivation by including only horses that possessed a certain basal level of food motivation and were willing to participate. Nonetheless, our results suggest that, in order to perform well in cognitive tasks involving learning, memory, and decision-making, such as inhibition tests, horses must be attentive to food rewards or prioritise food over other environmental stimuli to some degree.

### Reactivity is related to quick and flexible responding

The *Reactivity* component was additionally related to the two inhibitory control components *Indecisiveness* and *Flexibility*. Horses that scored high on the *Reactivity* component, scored lower on the *Indecisiveness* component. Accordingly, more reactive horses were quicker (short latency to make a choice in RL test) and more accurate in their reactions (accurate relearning in AB test and quick learning in food discrimination for DG test). At the same time, reactivity in the social coping context was related to higher flexibility in the inhibition tasks. However, no relationship was observable for the *Reactivity* scores in the food coping context. This suggests that either social cues are mediating this effect or rather that a stronger stressor is needed for observing this relationship. *Flexibility* was characterised by quick and flexible learning in the reversal learning and A-not-B task, while the *Indecisiveness* component might reflect slower decision-making but also generally slower processing of cognitive challenges and/or lower food motivation. Quick learning and flexible updating of novel task contingencies require low stress levels and a certain sensitivity to environmental stimuli (e.g.^86,87^). Horses showing a stronger stress sensitivity (i.e. low baseline cortisol and strong increase during acute stressor) made faster choices, were more accurate but also more flexibly in their behaviour. Similarly reactive pigs responded more flexible in a spatial discrimination task^13^ and reactive mice learned a task faster and were more attentive to changes in task contingencies^12^. Considering that one integral part of equitation is based on the ability to quickly and flexibly respond to the trainers’ cues^38^, our finding suggest that stress reactivity might indeed affect horses’ trainability.

### Limitations

While our study revealed small relations between coping behaviour and inhibitory control, it needs to be noted that several limitations need to be considered. First, we might not have completely captured inhibitory control in horses. While we selected three inhibitory control tests that have previously been established with horses and are thought to measure different aspects of inhibitory control, it is possible that we have missed aspects of inhibitory control in horses. A larger test battery with additional inhibitory control tests and a validation for temporal stability would be necessary to overcome this issue. Although, given the inherently flexible nature of IC, which requires a certain sensitivity to environmental stimuli for updating novel task contingencies, it may be difficult to demonstrate temporal stability of IC measures. Alternatively, it is possible that the two coping contexts (delayed turnout and delayed feeding) did not capture behaviours related to inhibitory control, but rather only how much the horses valued food or contact with conspecifics, rather than less context-specific coping behaviour. We selected those two contexts because they mimic situations that a horse could encounter on a daily basis; however, for example, we did not test the horses in situations, in which they would have more control over their exposure to the stressors, and thus more behavioural options to respond, such as novel situations (e.g. novel object/human test) or during interactions with humans (e.g. trainability) and conspecifics (i.e. dominance). Secondly, our sample population was rather standardised as we deliberately decided to reduce the variation of external influences for this first study on the relationship between coping and inhibitory control in horses. All horses were kept in suboptimal housing conditions (i.e. in individual stables with limited turnout), which have been linked to reduced welfare^35^. Consequently, it is possible that coping mechanisms had already been activated in response to the housing conditions and this was masking the relationship between coping and inhibitory control. In addition, the horses may have already learned that exhibiting certain behaviours would lead to faster success and, consequently, we did not capture their normal coping behaviour. For example, excessive kicking at the walls would prompt stable staff to feed these horses first or rearing and neighing would lead to them being turned out first. While we did not observe any changes in husbandry routine regardless of the horses’ behaviour, this could still have happened unconsciously. Testing more diverse horse populations in terms of sex, genetic background, training, and housing conditions may yield more nuanced results.

For future studies, it will be particularly interesting to investigate whether horses exhibiting high levels of stereotypical behaviours or horses that exhibit other signs of welfare impairment (e.g. apathy or aggression) show altered inhibitory control and whether this is modulated by the severity of the abnormal behaviours. Two horses in our sample exhibited intense crib-biting behaviour, which has been linked to dopamine dysfunction^88^ and altered impulse control and/or differently perceived reward values^89,90^,but see^44^. While these two horses did not show consistent differences in their scores derived from the inhibition tests compared to the other horses (except for both scoring very high on the *Indecisiveness* component), it is unclear whether they might have biased the results. Ultimately, it might be worthwhile to expand this line of research also to other farm animal species that often show abnormal behaviours due to suboptimal housing standards, such as pigs and chickens. Considering that inhibitory control can be enhanced by training^91^ and appears to have a strong genetic component^92^, new avenues for improving animal welfare may be particularly promising.

## Conclusion

In conclusion, we found that individual coping behaviour during mildly stressful husbandry conditions is partially linked to distinct aspects of inhibitory control capacity. Sensitivity to stress and attention to the environment seem to play a central part in horses’ behavioural inhibition, learning performance and cognitive flexibility.

## Supplementary Information

Below is the link to the electronic supplementary material.

Supplementary Material 1

## Data Availability

The raw data and the R code are available at: https:/osf.io/bws9d.

## References

[1] Dingemanse, N. J. & Wolf, M. Between-individual differences in behavioural plasticity within populations: causes and consequences. *Anim. Behav.* **85**, 1031–1039 (2013).

[2] Mason, G. et al. Plastic animals in cages: Behavioural flexibility and responses to captivity. *Anim. Behav.* **85**, 1113–1126 (2013).

[3] Koolhaas, J. M., de Boer, S. F., Buwalda, B. & van Reenen, K. Individual variation in coping with stress: A multidimensional approach of ultimate and proximate mechanisms. *Brain Behav. Evol.* 6–11. 10.1159/000105485 (2007). 10.1159/000105485 17914253

[4] Koolhaas, J. M., de Boer, S. F., Coppens, C. M. & Buwalda, B. Neuroendocrinology of coping styles: Towards understanding the biology of individual variation. *Front. Neuroendocr.* **31**, 307–321 (2010). 10.1016/j.yfrne.2010.04.001 20382177

[5] Øverli, Ø. et al. Evolutionary background for stress-coping styles: Relationships between physiological, behavioral, and cognitive traits in non-mammalian vertebrates. *Neurosci. Biobehav. Rev.* **31**, 396–412 (2007). 17182101 10.1016/j.neubiorev.2006.10.006

[6] Koolhaas, J. M. Coping style and immunity in animals: Making sense of individual variation. *Brain. Behav. Immun.* **22**, 662–667 (2008). 18395410 10.1016/j.bbi.2007.11.006

[7] Wechsler, B. Coping and coping strategies: a behavioural view. *Appl. Anim. Behav. Sci.* **43**, 123–134 (1995).

[8] Lea, S. E. G., Chow, P. K. Y., Leaver, L. A. & McLaren I. P. L. Behavioral flexibility: A review, a model, and some exploratory tests. *Learn. Behav.* **48**, 173–187 (2020). 32043268 10.3758/s13420-020-00421-w PMC7082303

[9] Coppens, C. M., Boer, S. F. D., Koolhaas, J. M., De Boer, S. F. & Koolhaas, J. M. Coping styles and behavioural flexibility: Towards underlying mechanisms. *Philosophical Trans. Royal Soc. B: Biol. Sci.* **365**, 4021–4028 (2010). 10.1098/rstb.2010.0217 PMC2992750 21078654

[10] Dalley, J. W., Cardinal, R. N. & Robbins, T. W. Prefrontal executive and cognitive functions in rodents: Neural and neurochemical substrates. *Neurosci. Biobehav. Rev.* **28**, 771–784 (2004). 15555683 10.1016/j.neubiorev.2004.09.006

[11] Benus, R. F., Koolhaas, J. M. & Van Oortmerssen, A. Aggression and adaptation to the light-dark cycle: role of intrinsic and extrinsic control. *Physiol. Behavior*. **43**, 131–137 (1988). 10.1016/0031-9384(88)90228-4 3212047

[12] Benus, R. F., Daas, D., Koolhaas, S., Van Oortmerssen, G. A. & J. M. & Routine formation and flexibility in social and non-social behaviour of aggressive and non-aggressive male mice. *Behaviour* **112**, 176–193 (1990).

[13] Bolhuis, J. E., Schouten, W. G. P., de Leeuw, J. A., Schrama, J. W. & Wiegant, V. M. Individual coping characteristics, rearing conditions and behavioural flexibility in pigs. *Behav. Brain. Res.* **152**, 351–360 (2004). 15196803 10.1016/j.bbr.2003.10.024

[14] Sih, A. & Del Giudice, M. Linking behavioural syndromes and cognition: a behavioural ecology perspective. *Philosophical Trans. Royal Soc. B-Biological Sci.* **367**, 2762–2772 (2012). 10.1098/rstb.2012.0216 PMC3427552 22927575

[15] Loyant, L., Collins, L. & Joly, M. Inhibitory control tests in non-human animals: validity, reliability, and perspectives. *Biol. Rev*. **70055**10.1111/brv.70055 (2025). 10.1111/brv.70055 PMC12586309 40717589

[16] Beran, M. J. The comparative science of ‘self-control’: what are we talking about? *Front. Psychol.* **6**, 51 (2015). 25688226 10.3389/fpsyg.2015.00051 PMC4311604

[17] Johnson-Ulrich, L. & Holekamp, K. E. Group size and social rank predict inhibitory control in spotted hyaenas. *Anim. Behav.* **160**, 157–168 (2020).

[18] Sheng, K., Foris, B., Krahn, J., Weary, D. M. & Von Keyserlingk, M. A. G. Redefining dominance calculation: Increased competition flattens the dominance hierarchy in dairy cows. *J. Dairy Sci.* **107**, 7286–7298 (2024). 38825128 10.3168/jds.2023-24587

[19] Diamond, A. Executive functions. *Annu. Rev. Psychol.* **64**, 135–168 (2013). 23020641 10.1146/annurev-psych-113011-143750 PMC4084861

[20] Moffitt, T. E. et al. A gradient of childhood self-control predicts health, wealth, and public safety. *Proc. Natl. Acad. Sci. U.S.A.* **108**, 2693–2698 (2011). 21262822 10.1073/pnas.1010076108 PMC3041102

[21] Robson, D. A., Allen, M. S. & Howard, S. J. Self-regulation in childhood as a predictor of future outcomes: A meta-analytic review. *Psychol. Bull.* **146**, 324–354 (2020). 31904248 10.1037/bul0000227

[22] Watts, T. W., Duncan, G. J. & Quan, H. Revisiting the marshmallow test: A conceptual replication investigating links between early delay of gratification and later outcomes. *Psychol. Sci.* **29**, 1159–1177 (2018). 29799765 10.1177/0956797618761661 PMC6050075

[23] Beran, M. J. & Hopkins, W. D. Self-control in chimpanzees relates to general intelligence. *Curr. Biol.* **28**, 1–6 (2018). 29429613 10.1016/j.cub.2017.12.043 PMC5820157

[24] Cervantes, M. C. & Delville, Y. Individual differences in offensive aggression in golden hamsters: A model of reactive and impulsive aggression? *Neuroscience* **150**, 511–521 (2007). 17964736 10.1016/j.neuroscience.2007.09.034

[25] Benus, R. F., Koolhaas, J. M. & Van Oortmerssen, G. A. Individual differences in behavioural reaction to a changing environment in mice and rats. *Behav* **100**, 105–121 (1987).

[26] Coppens, C. M., de Boer, S. F., Buwalda, B. & Koolhaas, J. M. Aggression and aspects of impulsivity in wild-type rats. *Aggressive Behav.* **40**, 300–308 (2014). 10.1002/ab.21527 24464354

[27] Gobbo, E. & Zupan Šemrov, M. Dogs exhibiting high levels of aggressive reactivity show impaired self-control abilities. *Front. Veterinary Sci.* **9**, 1–10 (2022). 10.3389/fvets.2022.869068 PMC8987203 35400110

[28] Overduin-de Vries, A. M., Vermande, M. M., Hessen, D. J. & Sterck, E. H. M. The ability to inhibit impulses is related to social behavior in long-tailed macaques. *Am. J. Primatol.*10.1002/ajp.23587 (2023). 38145328 10.1002/ajp.23587

[29] Garnham, L. C., Clarke, C. & Løvlie, H. How inhibitory control relates to positive and negative affective states in red junglefowl. *Front. Veterinary Sci.* **9**, 1–11 (2022). 10.3389/fvets.2022.872487 PMC9024352 35464350

[30] Garner, J. P. & Mason, G. J. Evidence for a relationship between cage stereotypies and behavioural disinhibition in laboratory rodents. *Behav. Brain. Res.* **136**, 83–92 (2002). 12385793 10.1016/s0166-4328(02)00111-0

[31] Garner, J. P., Mason, G. J. & Smith, R. Stereotypic route-tracing in experimentally caged songbirds correlates with general behavioural disinhibition. *Anim. Behav.* **66**, 711–727 (2003).

[32] Brucks, D., Marshall-Pescini, S., Wallis, L. J., Huber, L. & Range, F. Measures of dogs’ inhibitory control abilities do not correlate across tasks. *Front. Psychol.* **8**, 849 (2017). 28596749 10.3389/fpsyg.2017.00849 PMC5443147

[33] Loyant, L., Waller, B. M., Micheletta, J. & Joly, M. Validation of a battery of inhibitory control tasks reveals a multifaceted structure in non-human primates. *PeerJ* **10**, e12863 (2022). 35186469 10.7717/peerj.12863 PMC8840138

[34] Völter, C. J., Tinklenberg, B., Call, J. & Seed, A. M. Comparative psychometrics: Establishing what differs is central to understanding what evolves. *Philosophical Trans. Royal Soc. B: Biol. Sciences* **373**, (2018). 10.1098/rstb.2017.0283 PMC6107573 30104428

[35] Ruet, A. et al. Housing horses in individual boxes is a challenge with regard to welfare. *Animals* **9**, 1–19 (2019). 10.3390/ani9090621 PMC6770668 31466327

[36] Sarrafchi, A. & Blokhuis, H. J. Equine stereotypic behaviors: Causation, occurrence, and prevention. *J. Veterinary Behavior: Clin. Appl. Res.* **8**, 386–394 (2013).

[37] Lesimple, C. Indicators of Horse Welfare: State-of-the-Art. *Animals* **10**, 294 (2020). 32069888 10.3390/ani10020294 PMC7070675

[38] McBride, S. D. & Mills, D. S. Psychological factors affecting equine performance. *BMC Veterinary Research* **8**, (2012). 10.1186/1746-6148-8-180 PMC3514365 23016987

[39] Rankins, E. M. & Wickens, C. L. A systematic review of equine personality. *Appl. Anim. Behav. Sci.* **231**, 105076 (2020).

[40] Budzyńska, M. Stress Reactivity and Coping in Horse Adaptation to Environment. *J. Equine Veterinary Sci.* **34**, 935–941 (2014).

[41] Nagy, K., Bodó, G., Bárdos, G., Bánszky, N. & Kabai, P. Differences in temperament traits between crib-biting and control horses. *Appl. Anim. Behav. Sci.* **122**, 41–47 (2010).

[42] Fureix, C. et al. Plasma cortisol and faecal cortisol metabolites concentrations in stereotypic and non-stereotypic horses: do stereotypic horses cope better with poor environmental conditions ? (2013). 10.1186/1746-6148-9-3 PMC3544618 23289406

[43] Bachmann, I., Bernasconi, P., Herrmann, R., Weishaupt, M. A. & Stauffacher, M. Behavioural and physiological responses to an acute stressor in crib-biting and control horses. *Appl. Anim. Behav. Sci.* **82**, 297–311 (2003).

[44] Briefer Freymond, S. et al. Stereotypic horses (Equus caballus) are not cognitively impaired. *Anim. Cogn.* **22**, 17–33 (2019). 30328528 10.1007/s10071-018-1217-8

[45] Budzyńska, M., Kamieniak, J., Marciniak, B. & Sołtys, L. Relationships between thoroughbreds’ contribution in the pedigree and the level of fearfulness and performance in warmblood stallions. *Acta Vet.* **68**, 288–300 (2018).

[46] Hausberger, M., Richard-Yris, M. A. & Ricard, A. Interplay between environmental and genetic factors in the behaviour of horses. in Horse behaviour and welfare (Wagening Academic, The Netherlands, (2007).

[47] Osthaus, B., Proops, L., Hocking, I. & Burden, F. Spatial cognition and perseveration by horses, donkeys and mules in a simple A-not-B detour task. *Anim. Cogn.* **16**, 301–305 (2013). 23271641 10.1007/s10071-012-0589-4

[48] Brucks, D. & Härterich, A. König von Borstel, U. Horses wait for more and better rewards in a delay of gratification paradigm. *Front. Psychol.* **954472**10.3389/fpsyg.2022.954472 (2022). 10.3389/fpsyg.2022.954472 PMC9355425 35936272

[49] Rørvang, M. V., Nielsen, B. L. & McLean, A. N. Sensory abilities of horses and their importance for equitation science. *Front. Veterinary Sci.* **7**, 1–17 (2020). 10.3389/fvets.2020.00633 PMC7509108 33033724

[50] Brucks, D. et al. Intra – and interspecific variation in self – control capacities of parrots in a delay of gratification task. *Anim. Cogn.*10.1007/s10071-021-01565-6 (2021). 34671864 10.1007/s10071-021-01565-6 PMC8940755

[51] Alessandroni, N., Miller, R. & Altschul, D. Flexible behavior or flexible methods? A cross-taxon review of experimental designs in reversal learning. *Preprint at.*10.31234/osf.io/mvche_v2 (2025).

[52] Safryghin, A., Hebesberger, D. V. & Wascher, C. A. F. Testing for behavioral and physiological responses of domestic horses (Equus caballus) across different contexts - consistency over time and effects of context. *Front. Psychol.* **10**, 1–12 (2019). 31057468 10.3389/fpsyg.2019.00849 PMC6482254

[53] Young, T., Creighton, E., Smith, T. & Hosie, C. A novel scale of behavioural indicators of stress for use with domestic horses. *Appl. Anim. Behav. Sci.* **140**, 33–43 (2012).

[54] Peeters, M., Sulon, J., Beckers, J. F., Ledoux, D. & Vandenheede, M. Comparison between blood serum and salivary cortisol concentrations in horses using an adrenocorticotropic hormone challenge. *Equine Vet. J.* **43**, 487–493 (2011). 21496072 10.1111/j.2042-3306.2010.00294.x

[55] Contreras-Aguilar, M. D. et al. Effect of food contamination and collection material in the measurement of biomarkers in saliva of horses. *Res. Vet. Sci.* **129**, 90–95 (2020). 31954319 10.1016/j.rvsc.2020.01.006

[56] Vincent, A. et al. Evaluation of a modified bit device to obtain saliva samples from horses. *Veterinary Sciences* **8**, (2021). 10.3390/vetsci8100232 PMC8538290 34679064

[57] Palme, R. & Möstl, E. Measurement of cortisol metabolites in faeces of sheep as a parameter of cortisol concentration in blood. *Z Saugetierkd – Int J. Mammal Biol*. **62**, 192–197 (1997). Suppl. 2.

[58] Schmidt, A. et al. Cortisol release and heart rate variability in horses during road transport. *Horm. Behav.* **57**, 209–215 (2010). 19944105 10.1016/j.yhbeh.2009.11.003

[59] ASAB Ethical Committee/ABS Animal Care Committee. Guidelines for the ethical treatment of nonhuman animals in behavioural research and teaching. *Anim. Behav.* **195**, I–XI (2023).

[60] Percie, D. et al. The ARRIVE guidelines 2.0: Updated guidelines for reporting animal research. *PLoS Biol.* **18**, e3000410 (2020). 32663219 10.1371/journal.pbio.3000410 PMC7360023

[61] R Core Team. *R: A Language and Environment for Statistical Computing. R Foundation for Statistical Computing, Vienna, Austria* (R Foundation for Statistical Computing, 2021).

[62] Gamer, M., Lemon, J., Fellows, I. & Singh, P. Various Coefficients of Interrater Reliability and Agreement. (2019).

[63] Koo, T. K. & Li, M. Y. A Guideline of selecting and reporting intraclass correlation coefficients for reliability research. *J. Chiropr. Med.* **15**, 155–163 (2016). 27330520 10.1016/j.jcm.2016.02.012 PMC4913118

[64] Nagy, K., Bodó, G., Bárdos, G., Harnos, A. & Kabai, P. The effect of a feeding stress-test on the behaviour and heart rate variability of control and crib-biting horses (with or without inhibition). *Appl. Anim. Behav. Sci.* **121**, 140–147 (2009).

[65] Visser, E. K., Ellis, A. D. & Van Reenen, C. G. The effect of two different housing conditions on the welfare of young horses stabled for the first time. *Appl. Anim. Behav. Sci.* **114**, 521–533 (2008).

[66] Bates, D., Maechler, M., Bolker, B. & Walker, S. Fitting linear mixed-effects models using lme4. *J. Stat. Softw.* **67**, 1–48 (2015).

[67] Hartig, F. & DHARMa Residual Diagnostics for hierarchical (Multi-Level / Mixed) Regression Models. R package version 0.3.3.0 (2020).

[68] Baayen, R. *Analyzing Linguistic Data: A Practical Introduction to Statistics Using R* (Cambridge University Press, 2008).

[69] Roche, D. G., Careau, V. & Binning, S. A. Demystifying animal ‘personality’ (or not): why individual variation matters to experimental biologists. *J. Experiment. Biol. *146712 (2016). 10.1242/jeb.146712 10.1242/jeb.146712 27852750

[70] Raoult, C. M. C., Osthaus, B., Hildebrand, A. C. G., McElligott, A. G. & Nawroth, C. Goats show higher behavioural flexibility than sheep in a spatial detour task. *Royal Soc. Open. Sci.* **8**, (2021). 10.1098/rsos.201627 PMC8074883 33959332

[71] Brucks, D., Marshall-Pescini, S. & Range, F. Dogs and wolves do not differ in their inhibitory control abilities in a non-social test battery. *Anim. Cogn.* **22**, 1–15 (2018). 30284077 10.1007/s10071-018-1216-9 PMC6326967

[72] van Horik, J. O. et al. Do detour tasks provide accurate assays of inhibitory control? *Proceedings of the Royal Society B: Biological Sciences* 285, (2018). 10.1098/rspb.2018.0150 PMC5897648 29593115

[73] Vernouillet, A. A. A., Stiles, L. R., McCausland, J. A. & Kelly, D. M. Individual performance across motoric self-regulation tasks are not correlated for pet dogs. *Learning Behavior*. **46**, 522–536 (2018). 30251102 10.3758/s13420-018-0354-x

[74] Boogert, N. J., Anderson, R. C., Peters, S., Searcy, W. & Nowicki, S. Song repertoire size in male song sparrows correlates with detour reaching, but not with other cognitive measures. *Anim. Behav.* **81**, 1209–1216 (2011).

[75] Ducatez, S., Audet, J. N. & Lefebvre, L. Speed–accuracy trade-off, detour reaching and response to PHA in Carib grackles. *Anim. Cogn.* **22**, 625–633 (2019). 30929104 10.1007/s10071-019-01258-1

[76] Baragli, P., Vitale, V., Banti, L. & Sighieri, C. Effect of aging on behavioural and physiological responses to a stressful stimulus in horses (Equus caballus). *Behav* **151**, 1513–1533 (2014).

[77] Finy, M. S., Bresin, K., Korol, D. L. & Verona, E. Impulsivity, risk taking, and cortisol reactivity as a function of psychosocial stress and personality in adolescents. *Dev. Psychopathol.* **26**, 1093–1111 (2014). 24713465 10.1017/S0954579414000212

[78] Lyons, D. M., Lopez, J. M. & Yang, C. Schatzberg, a F. Stress-level cortisol treatment impairs inhibitory control of behavior in monkeys. *J. neuroscience: official J. Soc. Neurosci.* **20**, 7816–7821 (2000). 10.1523/JNEUROSCI.20-20-07816.2000 PMC6772868 11027246

[79] van Horik, J. O. & Madden, J. R. A problem with problem solving: Motivational traits, but not cognition, predict success on novel operant foraging tasks. *Anim. Behav.* **114**, 189–198 (2016). 27122637 10.1016/j.anbehav.2016.02.006 PMC4833691

[80] Velázquez-Sánchez, C. et al. High Trait impulsivity predicts food addiction-like behavior in the rat. *Neuropsychopharmacol* **39**, 2463–2472 (2014). 10.1038/npp.2014.98 PMC4138758 24776685

[81] Shaw, R. C. Testing cognition in the wild: factors affecting performance and individual consistency in two measures of avian cognition. *Behav. Process.* **134**, 31–36 (2017). 10.1016/j.beproc.2016.06.004 27288883

[82] van Horik, J. O. et al. Unpredictable environments enhance inhibitory control in pheasants. *Anim. Cogn.* **22**, 1105–1114 (2019). 31471781 10.1007/s10071-019-01302-0 PMC6834925

[83] Phelipon, R. et al. Characterisation of facial expressions and behaviours of horses in response to positive and negative emotional anticipation using network analysis. *Preprint at.*10.1101/2025.02.02.636153 (2025). 10.1371/journal.pone.0319315 PMC12077714 40367029

[84] Ricci-Bonot, C. & Mills, D. S. Recognising the facial expression of frustration in the horse during feeding period. *Appl. Anim. Behav. Sci.* **265**, 105966 (2023).

[85] Cooper, J. J., Mcall, N., Johnson, S. & Davidson, H. P. B. The short-term effects of increasing meal frequency on stereotypic behaviour of stabled horses. *Appl. Anim. Behav. Sci.* **90**, 351–364 (2005).

[86] Bebus, S. E., Small, T. W., Jones, B. C., Elderbrock, E. K. & Schoech, S. J. Associative learning is inversely related to reversal learning and varies with nestling corticosterone exposure. *Anim. Behav.* **111**, 251–260 (2016).

[87] Ruiz-Gomez, M. D. L., Huntingford, F. A., Øverli, Ø., Thörnqvist, P. O. & Höglund, E. Response to environmental change in rainbow trout selected for divergent stress coping styles. Physiol. Behav. **102**, 317–322 (2011). 21130105 10.1016/j.physbeh.2010.11.023

[88] McBride, S. D. & Hemmings, A. Altered mesoaccumbens and nigro-striatal dopamine physiology is associated with stereotypy development in a non-rodent species. *Behav. Brain. Res.* **159**, 113–118 (2005). 15795004 10.1016/j.bbr.2004.10.014

[89] McBride, S. D., Roberts, K., Hemmings, A. J., Ninomiya, S. & Parker, M. O. The impulsive horse: Comparing genetic, physiological and behavioral indicators to those of human addiction. *Physiol. Behav*. **254**, 113896 (2022). 35777460 10.1016/j.physbeh.2022.113896

[90] Parker, M., Redhead, E. S., Goodwin, D. & McBride, S. D. Impaired instrumental choice in crib-biting horses (Equus caballus). *Behav. Brain. Res.* **191**, 137–140 (2008). 18430476 10.1016/j.bbr.2008.03.009

[91] Smith, T., Panfil, K., Bailey, C. & Kirkpatrick, K. Cognitive and behavioral training interventions to promote self-control. *J. Experimental Psychology: Anim. Learn. Cognition*. **45**, 259–279 (2019). 10.1037/xan0000208 PMC6716382 31070430

[92] Friedman, N. P. et al. Individual differences in executive functions are almost entirely genetic in origin. *J. Exp. Psychol. Gen.* **137**, 201–225 (2008). 18473654 10.1037/0096-3445.137.2.201 PMC2762790

