# Supplementary material for: Exploring the relationship between inhibitory control and coping behaviour in horses
Source: Sci Rep. 2026 Apr 18;16:12738. doi: 10.1038/s41598-026-48050-z (PMC13091911; doi:10.1038/s41598-026-48050-z)
Supplement: Supplementary file 1 — Supplementary Material 1 [file 41598_2026_48050_MOESM1_ESM.pdf]

# Supplementary Material

## Exploring the relationship between inhibitory control and coping behaviour in horses

Marie von der Tann[1], Rupert Palme[2], Uta König von Borstel[3] & Désirée Brucks[1*]

[1] Animal Husbandry & Ethology, Albrecht Daniel Thaer-Institute of Agricultural and Horticultural Sciences, Faculty of Life Sciences, Humboldt-Universität zu Berlin, Germany

[2] Department of Biological Sciences and Pathobiology, University of Veterinary Medicine, Vienna, Austria

[3] Department of Animal Breeding and Genetics, University of Giessen, Germany

## Supplementary Methods

### Inhibitory Control Test Battery

For all inhibition tests, we used pieces of carrots as rewards. Since most horses had not been fed carrots prior to the study, we habituated the horses to eating carrots by mixing them with their daily concentrate feed. We only started testing, if a horse reliable took pieces of carrot from the hand. The order of tests was randomly assigned to each horse (see Table S1).

**Table S1.** Sequences of inhibition tests

| Number of horses assigned | 1st test | 2nd test | 3rd test |
|---|---|---|---|
| **6** | DG | AB | RL |
| **5** | AB | DG | RL |
| **4** | RL | AB | DG |
| **5** | AB | RL | DG |
| **6** | RL | DG | AB |
| **5** | DG | RL | AB |

AB = A-not-B Test; DG = Delay of Gratification Test; RL = Reversal Learning Test

#### *A-not-B Test*

The A-not-B Test was conducted outside on a designated individual sand paddock (10 x 13 m) with a camera on a tripod positioned next to the paddock to capture the horse's behaviour. Horses were only tested when the rest of the horses from that stable were also outside on the adjacent paddocks to reduce separation anxiety.

An assistant led the horse from the box on to the paddock using a rope attached to the halter and released the horse to freely explore the paddock (10 x 13 m) for 1-2 mins. The jumping posts and rails that were used as hurdles later on were positioned on the side of the paddock, so that the horse could see and investigate the material without experiencing the physical barrier yet. Following this, the horse was habituated to feeding from a bucket (green plastic bucket 60 l volume, 60 cm height) and to associate the bucket with a food reward (see Table S2). The assistant collected the horse and led her to the edge of the paddock. The experimenter

took a hand full of carrot pieces and visible dropped these into the green bucket, then shook the bucket and placed it on the ground approximately 1 m in front of the horse. The horse was released and could eat the carrots. If the horse fed within 10 s, she was collected again and positioned at the edge of the paddock. The bucket was placed at a different position each time, to ensure that the horse was comfortable to approach the bucket at different positions on the paddock. If the horse did not approach the bucket or showed signs of fear, the bucket was lifted, shown to her for inspection and she was allowed to feed from the bucket when held at breast height. Once the horse fed from the bucket, the next step was initiated and the bucket was visibly baited and placed at consecutively larger distances to the horse (2 m, 3 m). If a horse successfully (i.e. within 10 s after placing it on the ground) approached the bucket at the final distance of 3 m, the horse was collected again and led to a corner of the paddock. The experimenter then built up the hurdles consisting of two jumping hurdles with a single rail on each post, leaving a gap of 1.5 m on both sides. Pilot testing revealed that the horses had problems to detour hurdles and did not try to find a solution, consequently, we implemented a training phase, in which the horses were familiarised with the hurdles and the possibility to detour around. The assistant led the horse around the hurdles once to each side before positioning the horse again at the edge of the paddock. The experimenter then shifted the hurdles to close the gap on one side, so that only one gap remained for the test. The side of the gap was randomly assigned for each horse by alternately assigning horses to either side based on an alphabetically ordered list of all horses' names.
In case that a horse showed signs of distress (e.g. excessive running, neighing), the session was terminated and repeated the next day. A maximum of 12 trials were performed per session and if the horse did not reach the criterion, the session was repeated the next day. If a horse did not find the gap in the first five trials, the assistant led the horse to the gap and towards the food bucket once (N = 9 horses required help). If the criterion was not reached within a maximum of 30 trials or 4 sessions, the test was terminated (N = 1 horse). In the B-phase, the gap was moved to the opposite side. The assistant positioned the horse at the edge of the paddock while the experimenter moved the hurdles.

**Table S2.** Overview test phases A-not-B test

| Order | Phase | Description | Trials | Criterion |
|---|---|---|---|---|
| 1 | Habituation | Association between bucket and food reward | --- | Feed from bucket at 3m distance |
| 2 | Demonstration | Leading horse around hurdles | --- | --- |
| 3 | A-trials | Learning to detour hurdles to get to food reward | Max. 30 | 3x crossing gap within 60 s in consecutive trials |
| 4 | B-trials | Switching side of gap, re-learning of detour behaviour | 5 | --- |

### *Delay of Gratification Test*

A robust nylon barrier was installed in the box door, which was subsequently opened. The experimenter was sitting on the ground at a distance of around 2 m from the box. In front of her, two plastic plates (gardening pot trays, diameter 30 cm) in white and black were positioned. A broom stick (60 cm length) was attached to each plate to allow the experimenter to move the

plates towards the horse. Pieces of carrots were placed on the plates. Depending on each horses' neck length and propensity to reach for the food, the plates were positioned at a distance of 1-1.5 m from the box. For fourteen horses, we needed to adjust the setting as they were too excited (and thus posed a risk to themselves and the experimenter). For these horses, the test was conducted on a table (1m height) instead of on the ground and their box window served as a barrier instead of the nylon barrier. The horses tested using the table-setup did not demonstrate any different waiting success compared to the horses that were tested on ground-level (LM: -0.475 ± 0.497, t = -0.956, p = 0.347).

To habituate the horse to the plates, we conducted three habituation trials, in which a piece of carrot was placed on the plate that was assigned to the high-quantity reward (HQR) and moved towards the horse. If the horse consumed the food within 5 s in three consecutive trials, the horses proceeded to the quantity discrimination phase (see Table S3 and main text). In the quantity discrimination test, the experimenter visibly placed the carrots on the respective plates (HQR and LQR) simultaneously and moved them slightly towards the horse at the same time. The plates remained in this intermediate position, in which the horse could not reach them for 2 s and then moved them within reach of the horse. The horse was allowed to choose one plate (i.e. touching the food counted as choice), while the non-chosen plate was immediately moved backwards out of reach again. If a horse did not select either option within 5 s, the trial was repeated and the next trial was initiated after an inter-trial interval of approximately 10 s. We conducted 12 trials per session with alternating sides of the HQR. Once horses clearly discriminated between LQR and HQR (at least 10 HQR choices out of 12 trials; binomial test: p = 0.038), they progressed to the training phase.

**Table S3.** Overview of test phases in delay of gratification test

| Order | Phase | Description | Trials | Criterion |
|---|---|---|---|---|
| 1 | Habituation | Feed from HQR plate | 3 | Feed from plate within 5s |
| 2 | Quantity discrimination test | Discriminate between quantities | 12 per session (max. 10 sessions (N = 0)) | 10/12 choices for HQR |
| 3 | Training | Learn contingencies: LQR immediate vs. HQR after 1s | 12 per session (max. 6 sessions (N = 5)) | 10/12 choices for HQR |
| 4 | Delay test | Wait for better reward with increasing delays: 2 s, 5 s, 10 s, 15 s, 20 s | 15 per session (max. 6 sessions per delay)<br>12 test trials w delay<br>3 control trials:<br>- HQR on both plates<br>- LQR on both plates<br>- Position control (reversed order = HQR 1st and LQR 2nd ) | 4/12 choices for HQR within one session in test trials in 2 consecutive sessions |

HQR = high-quantity reward; LQR = low-quantity reward

**Table S4.** Example of trial sequence in a delay of gratification test session

| *Trial* | *Trial Type* |
|---|---|
| *1* | Test |
| *2* | Test |
| *3* | LQR control |
| *4* | Test |
| *5* | Test |
| *6* | Test |
| *7* | HQR control |
| *8* | Test |
| *9* | Test |
| *10* | Test |
| *11* | Test |
| *12* | Test |
| *13* | Test |
| *14* | Test |
| *15* | Position control |

### *Reversal-Learning Test*

The RL test was conducted in front of the horse's box through a nylon barrier or through the box window (if the box had a window). The experimenters were standing in a central position in front of the horse with a distance of 1-1.5 m, again depending on the horse's neck length and propensity to reach over the barrier. The experimenter held the two stimuli in her hands and presented them to the horse by stretching the arms forward. This setting allowed adjusting for movements of the horse by positioning the experimenter and stimuli in a manner that always both stimuli were equidistant to the horse with a distance of 0.3 m between the two stimuli.

To familiarise the horses with the symbols and the test procedure, a habituation phase was conducted (see Table S5). The nylon barrier was installed, the experimenter and assistant were standing in front of the horse and only the assigned S+ symbol was presented to the horse. As soon as the horse touched the symbol with her nose, the experimenter verbally praised the horse, and the assistant stepped to the side and stretched out the arm with the plate and carrot on it. If a horse did not touch the symbol within 10s, the experimenter moved the symbol closer to the horse and rewarded any movement towards the symbol (verbal praise + food reward) until the horse made physical contact with the symbol. If a horse successfully touched the S+ symbol within 10s in three consecutive trials, the habituation was completed and the test phase started (see main text for description of acquisition and reversal phase).
In the test phase, the experimenter stretched out her arms with the two stimuli simultaneously, holding them in front of her for 1 s before stretching them forward within reach of the horse. The horse then had 20 s to make a choice (i.e. touch one stimulus with nose). If the horse chose S+, the experimenter praised verbally, lowered the S- stimuli, and the second experimenter stepped to the side and provided the food reward. If the horse chose S-, the experimenter lowered both stimuli and remained motionless until the next trial started after an inter-trial interval of 5 s. If no choice was made within 20 s, the trial was repeated.

**Table S5.** Overview of test phases in reversal learning test

| **Order** | **Phase** | **Description** | **Trials** | **Criterion** |
|---|---|---|---|---|
| 1 | Habituation | Touch S+ to get food reward | Until criterion reached | Touch S+ within 10s in 3 consecutive trials |
| 2 | Acquisition | Discriminate between S+ and S- | 12 per session (max. 20 sessions (N = 1)) | 10/12 choices for S+ |
| 3 | Reversal | Reversed contingencies: S+ is no longer rewarded, instead S- is rewarded | 12 per session (max. 20 sessions (N = 3)) | 10/12 choices for S- |

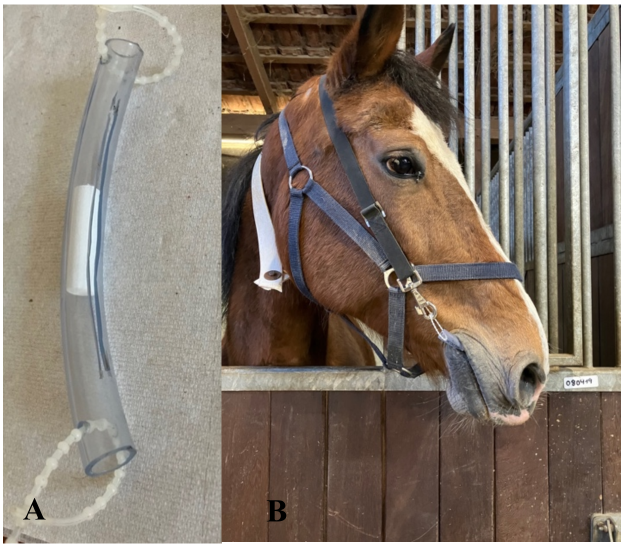


**Figure S1.** Saliva collection in horses: **A**) the swab was inserted in a PVC tube and **B**) placed in the horse's mouth.

## Supplementary Analyses

### Reliability coding

To assess reliability between coders, 20% of randomly selected videos of relevant sessions (excl. habituation and training phases) were coded by a second coder and we assessed the Intra-Class Correlation Coefficient (two-way, agreement; see Table S6) for all raw variables that were entered into the PCA using the *irr* package (Gamer et al., 2019).

**Table S6.** Intra-class correlation coefficients for reliability coding of variables that were used for the PCAs.

| **Variable** | **ICC (two-way, agreement)** |
|---|---|
| ***Inhibitory Control Tests*** | |
| AB latency gap | 0.999 |
| AB accuracy | 0.836 |
| DG choice | 0.957 |
| DG pawing | 0.774 |
| DG turn away | 0.745 |
| RL choice | 0.992 |
| RL latency | 0.976 |
| ***Coping Tests*** | |
| Aggression | 0.802 |

| | |
|---|---|
| Shake | 0.862 |
| Snort | 0.881 |
| Check trough | 0.899 |
| Rear | 0.994 |
| Defecate | 0.603 |
| Head movement | 0.875 |
| Nicker | 0.659 |
| Yawn | 0.912 |
| Locomotion | 0.970 |
| Alert | 0.786 |
| Feed | 0.974 |
| Pawing | 0.915 |
| Neigh | 0.970 |
| Groom | 0.808 |
| Bar-bite | 0.829 |
| Oral behaviours | 0.650 |

## Supplementary Results

***A-not-B Test***

To assess whether the AB Test captured the horses' inhibition capacities, we analysed the two main variables in separate models. The accuracy was analysed in a binomial GLMM (lme4 package (Bates et al., 2015)) using the *cbind*-function to set the number of accurate choice and inaccurate choices as response variable. Trial (factor: A1, A2, A3, B1, B2, B3, B4, B5) and side of the gap (factor: L, R) were included as predictors with horse ID and experimenter ID as random effects. To consider the upper limit of trial duration and the resulting right skewed distribution of latencies, we modelled the latency to cross the gap using a Proportional Hazards Model (*coxme*-function within coxme package (Therneau, 2020), and survminer package (Kassambara et al., 2020)) in which the latency was set as a censored response variable. As before, trial and side of gap were included as predictors and horse ID and experimenter ID as random effects. Additionally, trial was included as a random slope for horse ID.

One horse did not learn to detour around the hurdles within the maximum number of 30 trials and was not further tested in the A-not-B Test. All other horses learned to reliably detour the hurdles, reaching the A-criterion within a median of 6 trials (range = 3-23).

The horses' accuracy showed a clear drop in the first B-trial with a statistically supported effect of trial ($p < 0.001$; see Fig. S2a). The side of the gap did not affect the accuracy ($p = 0.290$; see Fig. S2b).

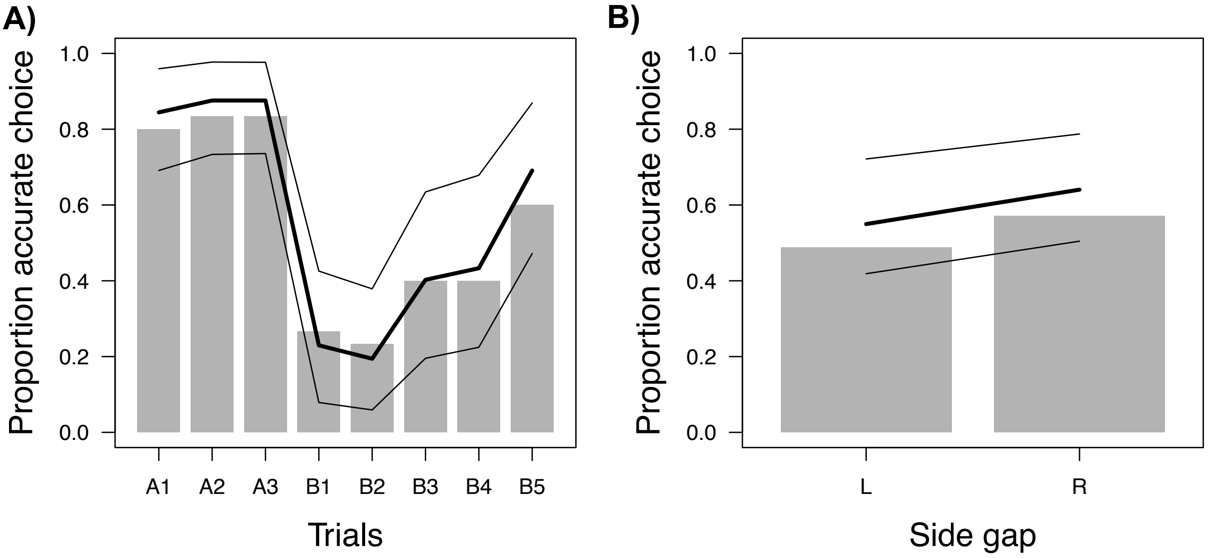


**Figure S2.** Proportion of horses with accurate choices in the A-not-B test as a function of **A**) trial and **B**) side of the gap. The bold black line depicts the fitted model and the outer lines the respective 95% confidence intervals.

For the latency, we found an effect of trial (LRT: $\chi^2{}_7 = 30.54$, $p < 0.001$) but no effect of the gap's side (LRT: $\chi^2{}_1 = 0.88$, $p = 0.348$). The horses showed an increased latency to cross the gap in the first B trial compared to all three A-trials (see Fig. S3a). Furthermore, horses got faster in finding the gap from the first A-trial to the third A-trial and reached a latency similar to the first A-trial from the third B-trial onwards. Whether the gap was located left or right, did not affect the latency (see Fig. S3b).

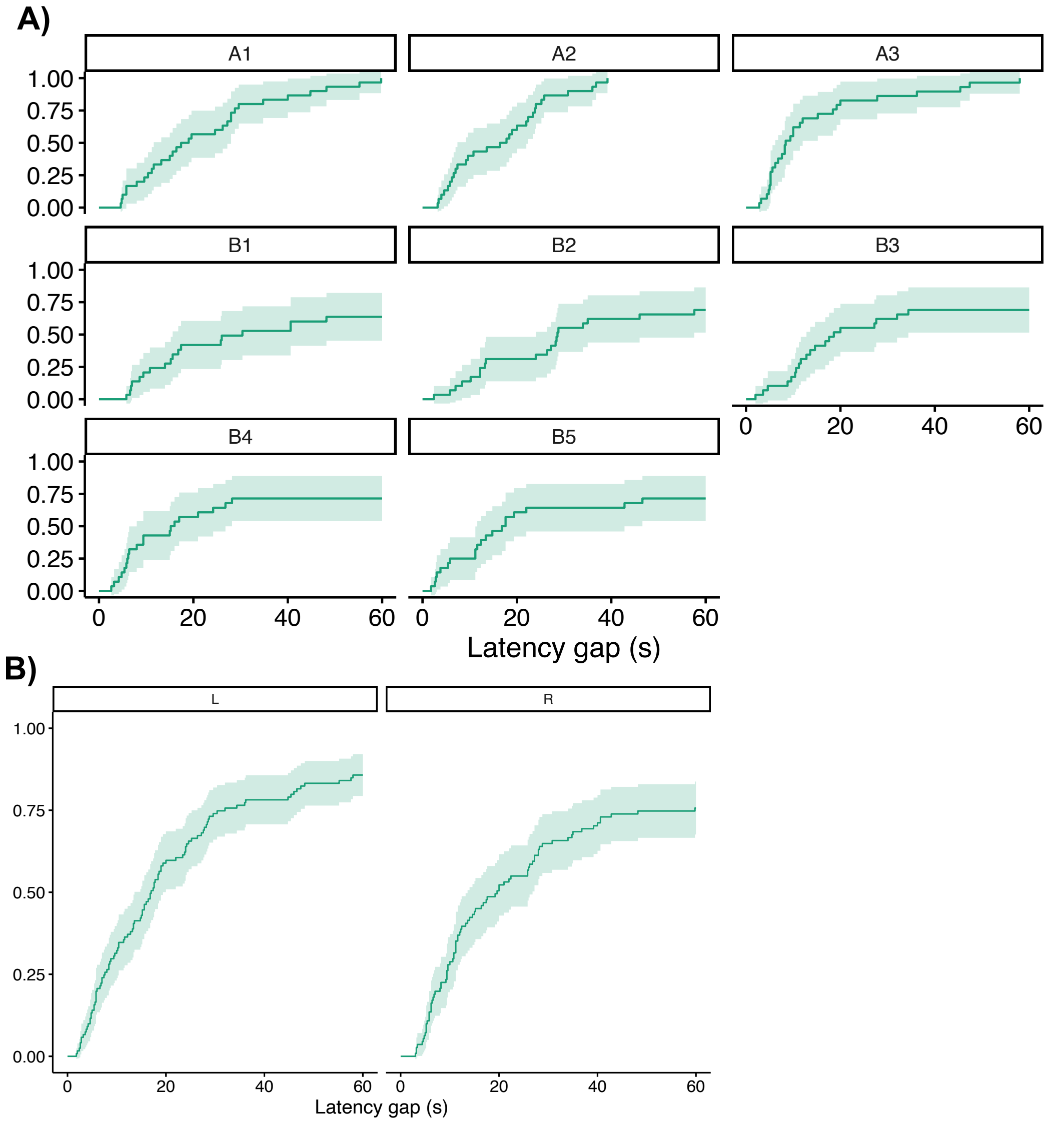


**Figure S3.** Cumulative incidence of crossing the gap plotted per **A**) trial and **B**) side of gap. The line depicts the estimated probability of succeeding. The shaded area shows the 95% confidence interval.

### ***Delay of Gratification Test***

To analyse the horses' behaviour in the DG test, we ran two models, one binomial GLMM with the number of choices for the 2$^{nd}$ plate (= correct choice) and the number of choices for the 1$^{st}$ plate (= incorrect choice) per session as response variable using the *cbind*-function and using only the test trials as data. Delay stage (z-transformed to a mean of 0 and a standard deviation of 1), session number per delay stage (z-transformed), proportion of waiting behaviours (summed waiting behaviours/trial duration), colour of the HQR plate (factor: black, white), as well as an interaction between delay x session and delay x proportion waiting behaviours were included as predictors. Horse ID, experimenter ID, and date were used as random effects with delay and session as random slopes for horse and experimenter ID. The second binomial GLMM was run including only the choices in the control trials, again using the *cbind*-function and number of choices for 2$^{nd}$ and 1$^{st}$ plate per session as response variable. Delay (z-

transformed), trial type (factor: LQR control, HQR control, Position control) and an interaction between the two as predictors. The same random effect structure as in the first model was used.

All horses passed the quantity discrimination test (session to criterion: median: 4; range: 1-10). Five horses did not pass the training phase with a 1s delay. Five horses reached the maximum delay of 20 s, while only three horses could successfully wait for 20 s (Fig. S4A). On a group level, the horses waited for a median of 2 s (range: 0-20 s).

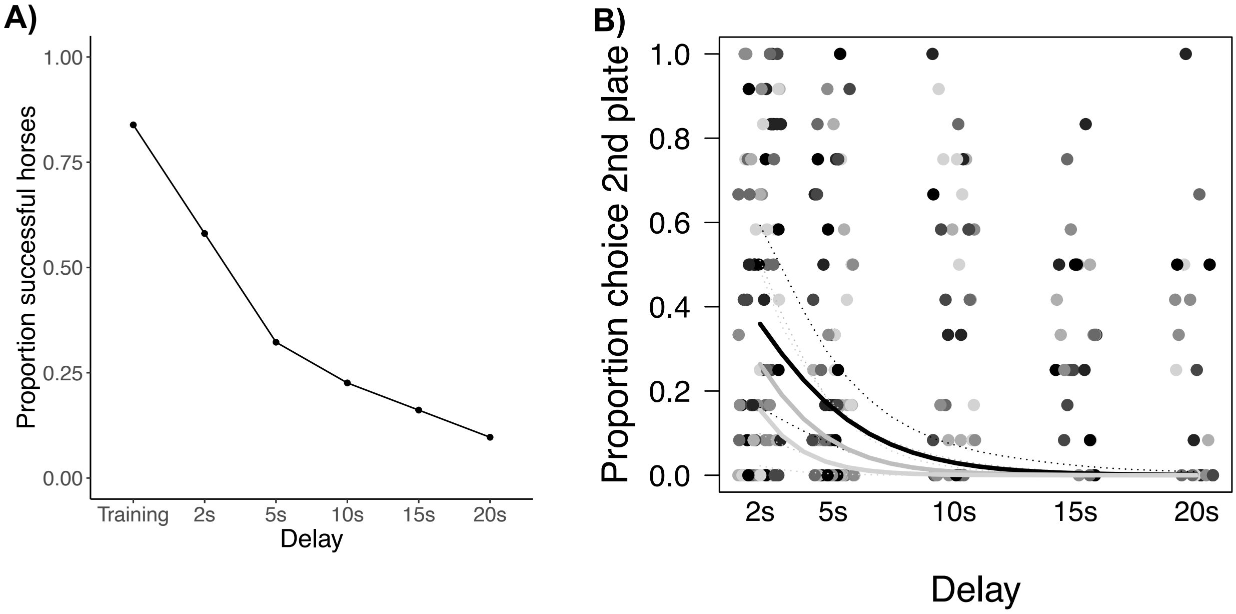


**Figure S4. A)** Proportion of successful horses per delay stage. **B)** Proportion of choosing the 2nd plate (= waiting for better reward) in test trials plotted per delay stage and test session. The darker the points, the earlier the session, the lighter the points, the later the session. The black line indicates the model estimates for the first test session across delay stages, the gray line indicates the model estimates for the 3rd test session, and the light gray line shows the estimates for the 6th test session. The dotted lines depict the 95 % confidence intervals.

*Test trials*

Horses' waiting success decreased with increasing delay and session number (LRT: $\chi^2_1$ = 18.964, p < 0.001; see Fig. S4B). We could not detect an interaction between delay and waiting behaviours (LRT: $\chi^2_1$ = 2.739, p = 0.098). As a main effect, waiting behaviours were positively associated with waiting success (p < 0.001; see Fig. S5B). Colour of the HQR plate did not affect waiting success (p = 0.383; see Fig. S5B).

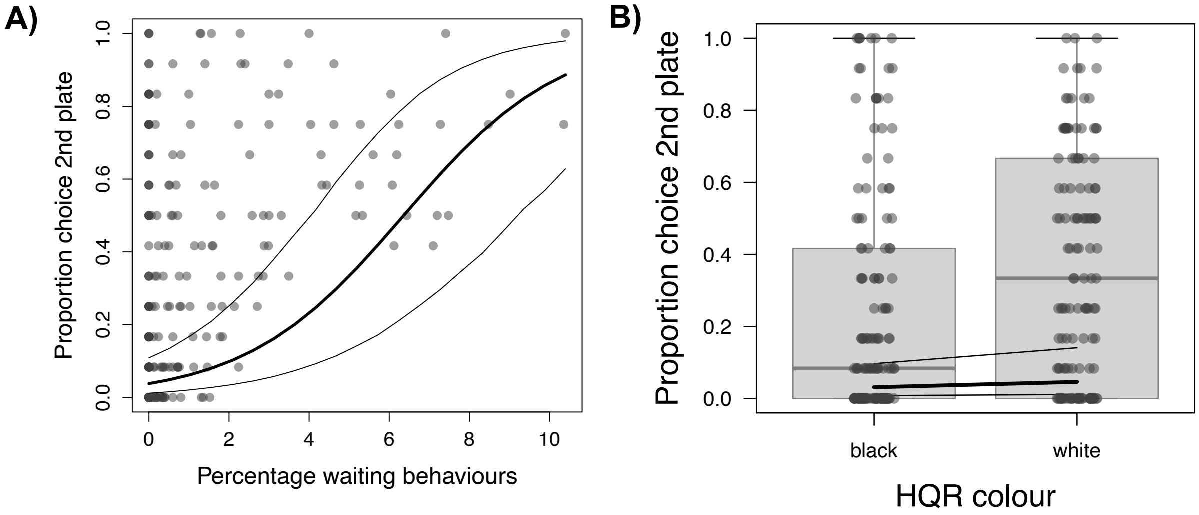


**Fig. S5.** Proportion of choices for 2nd option in test trials plotted **A)** against the percentage of waiting behaviours emitted per trial duration across all delay stages, and **B**) per colour used for HQR plate. The bold black line depicts the fitted model and the outer lines the respective 95% confidence intervals.

*Control trials*

In a separate model, we assessed the horses' choice behaviour in the three control trials. The horses clearly distinguished between the control trials (LRT: $\chi^2_2 = 107.960$, $p < 0.001$; Fig. S6). Horses never waited for the second option in the position control trials (HQR 1st / LQR 2nd; see Fig. S6A); thus, suggesting that the horses were paying attention to the content of plates and correctly identified that waiting does not pay off since the HQR was immediately available. Slightly higher waiting rates were observed in the HQR control (HQR presented on both plates; see Fig. S6A), which might indicate that the horses were focused on the 2nd option while avoiding the 1st option. In the LQR control trials (LQR presented on both plates), horses showed the highest waiting rate (see Fig. S6A) of all controls. This might be explained by the fact that the horses were indeed either avoiding the 1st option or were not able to perceive that the 2nd option contained only LQR instead of the expected HQR. We detected neither an effect of delay x control trial type interaction (LRT: $\chi^2_2 = 0.371$, $p = 0.831$) nor an effect of delay on choice behaviour in the control trials (LRT: $\chi^2_1 = 0.022$, $p = 0.883$; see Fig. S6B).

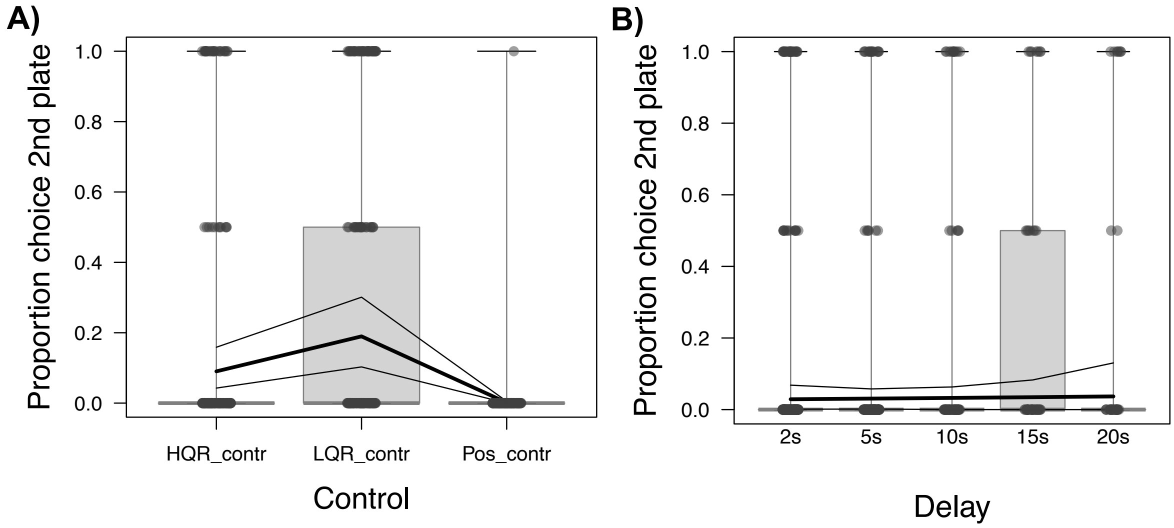


**Fig. S6.** Proportion of choices for 2nd option in control trials plotted **A)** per control type across all delay stages, and **B**) across delay stages. HQR_contr – both plates contained HQR; LQR_contr – both plates contained LQR; Pos_contr – reversed order of plates with HQR 1st and LQR 2nd. The bold black line depicts the fitted model and the outer lines the respective 95% confidence intervals.

***Reversal Learning Test***

To analyse the data from the RL test, we ran two models, one GLMM (binomial) to model the choices and one LMM to analyse the choice latency. The GLMM was used with the number of correct choice (*cbind* – function, with correct, incorrect choices) per session as response variable and session (z-transformed), phase (factor: acquisition, reversal), S+ symbol (factor: circle, square), and an interaction between session and phase as predictors. Horse ID, experimenter ID, and date were set as random effects with session, and phase as random slopes. For the LMM, we log-transformed the choice latency as response variable (on a trial basis) and used a three-way interaction between choice (binary), phase (factor), and session (z-transformed), as well as trial number (z-transformed) as response variables. Horse ID, experimenter ID and date were used as random slopes.

Horses needed a median of 5 sessions to reach the learning criterion in the acquisition phase (range: 1-20). One horse did not reach the learning criterion within the maximum number of 20 sessions and did not experience the reversal phase. The remaining 30 horses were tested in the reversal phase, however, two horses failed to reach the reversal criterion within 20 sessions and due to an experimenter mistake, two horses were tested with an incorrect S+ symbol in the reversal phase (in session 2 and 7 respectively). Testing was terminated upon realising the mistake. The twenty-four horses that reached the reversal criterion needed a median of 7.5 sessions (range: 2-20; Fig. S7).

Horses were less successful in the reversal phase compared to the acquisition phase ($p < 0.001$), thus, indicating that they had to inhibit their response based on the initial association and needed to reverse their response pattern (see Fig. S7). Furthermore, horses improved their success across sessions ($p < 0.001$; see Fig. S7). We could neither detect a session x phase interaction (LRT: $\chi^2_1 = 0.310$, $p = 0.578$) nor a phase x symbol interaction (LRT: $\chi^2_1 = 0.516$,

p = 0.473); however, horses were generally less successful with the square as S+ symbol compared to the circle (p = 0.004; see Fig. S7).

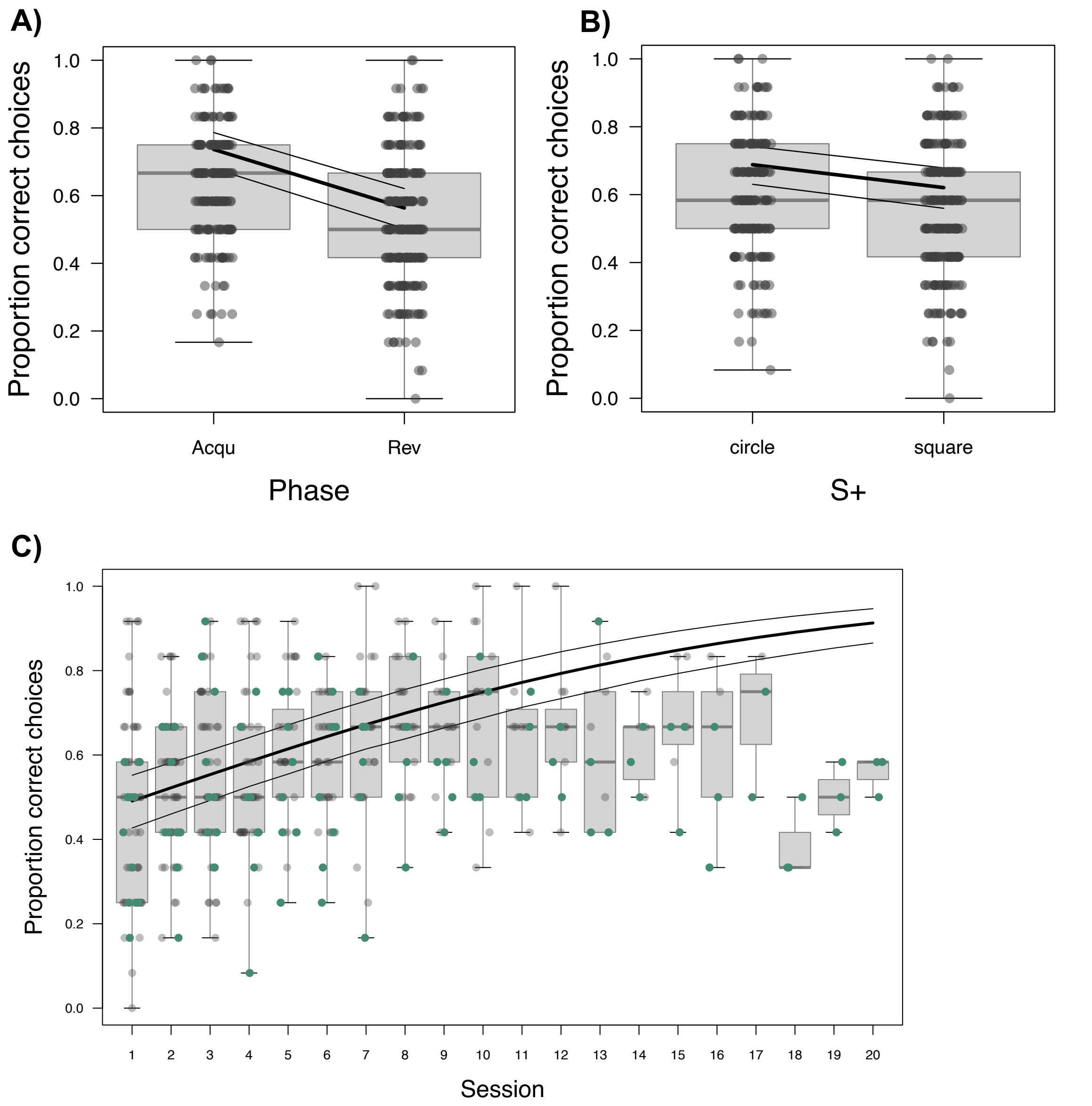


**Figure S7.** Proportion of correct choices as a function of **A**) test phase, **B**) S+ symbol, and **C)** session in the RL test. The thick black lines indicate the model estimates and the thinner black lines the 95 % confidence intervals. Points show individual data points. Green points indicate individual horses that did not reach the learning criterion.

For the latency to make a choice, we detected an effect of the three-way interaction between choice, phase, and session (p = 0.018; see Fig. S8). Horses got faster in making a choice across sessions in the acquisition phase irrespective of the choice, while in the reversal phase, horses only made faster choices across sessions, when choosing correctly (see Fig. S8). We found no detectable effect of trial number on the choice latency (LRT: $\chi^2_1 = 1.892$, p = 0.169).

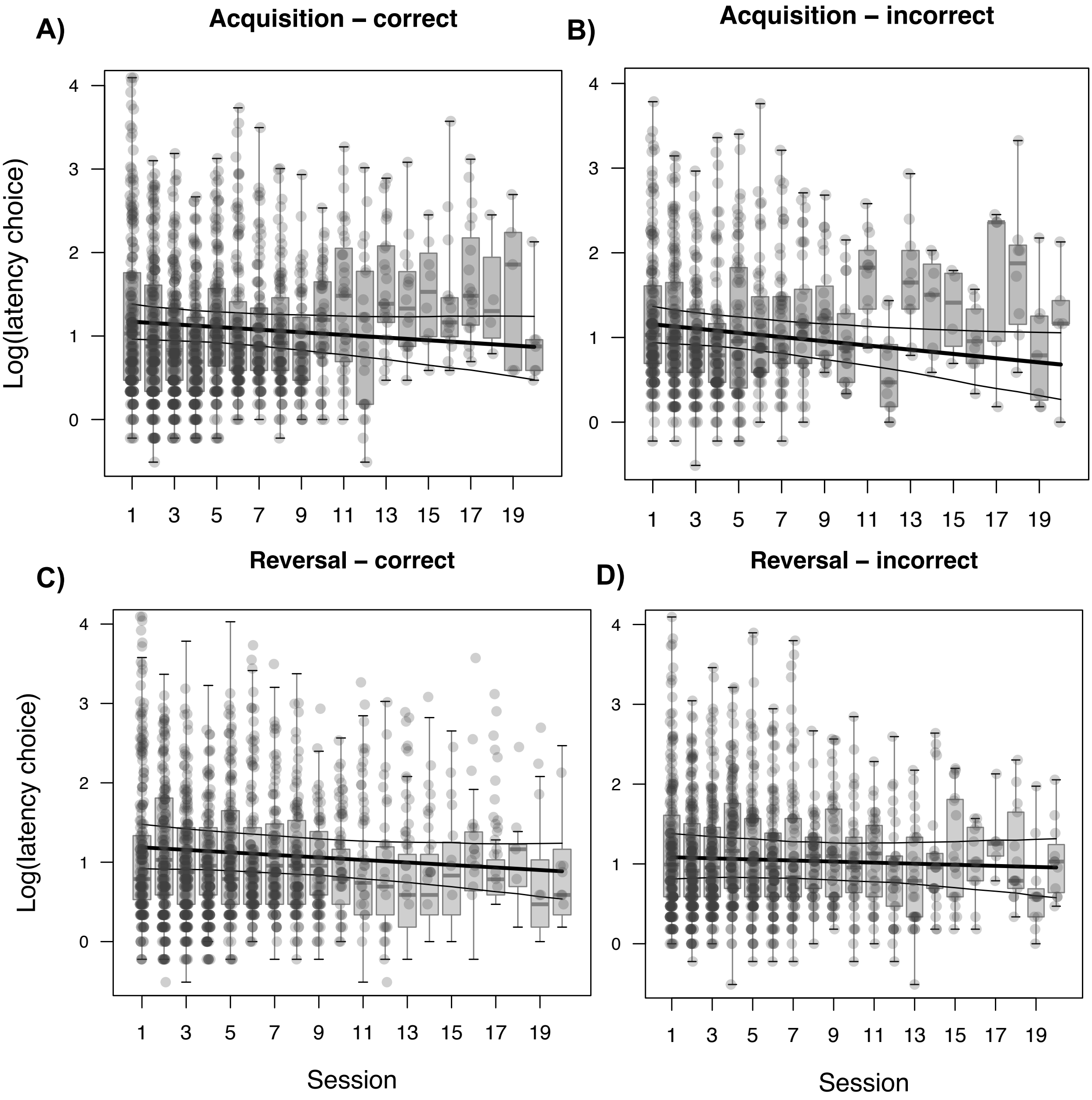


**Figure S8.** Latency to make a choice as a function of session number per **A)** acquisition phase with correct choices, **B)** acquisition phase with incorrect choices, **C)** reversal phase with correct choices, **D)** reversal phase with incorrect choices. Solid lines show model estimates, dotted lines depict 95 % confidence intervals. Points indicate individual data points.

**Cortisol measurements – complementary results**

Nine saliva samples could not be analysed as they contained too little saliva. These were five single samples from five different horses (4x baseline, 1x after treatment) and both samples from one horse in two social sessions.

We could not detect a session x context interaction (LRT: $\chi^2_1$ = 1.289, p = 0.256) and likewise no effect of session as a main effect (LRT: $\chi^2_1$= 0.034, p = 0.855), thus, indicating that the horses' reaction in the coping tests was temporally consistent. An interaction between

context and time point showed that higher cortisol concentrations were found in the 2nd sample compared to the baseline sample in the social coping context but not in the food coping context (see Fig. S9). The delayed feeding did not lead to an increase in salivary cortisol (see Fig. S9A), whereas the delayed turnout induced an increase in salivary cortisol (see Fig. S9B). Compared with stressors used in other studies (e.g. Massányi et al. (2023) – transport, shoeing, treadmill training: 3.59-4.16 ng/ml; Schmidt et al. (2010) – long-distance transport: 4.1-6.5 ng/ml), this increase is very moderate and might indicate that our coping tests did not elicit a strong physiological reaction, at least an average. The baseline cortisol measures (median: 0.68 ng/ml; range: 0.07 – 3.85 ng/ml) were within the range observed in other studies (0.8-1.2 ng/ml; Aurich et al., 2015)(0.6-0.65 ng/ml; Bohák et al., 2013; 0.28-0.42 ng/ml; Schmidt et al., 2010).

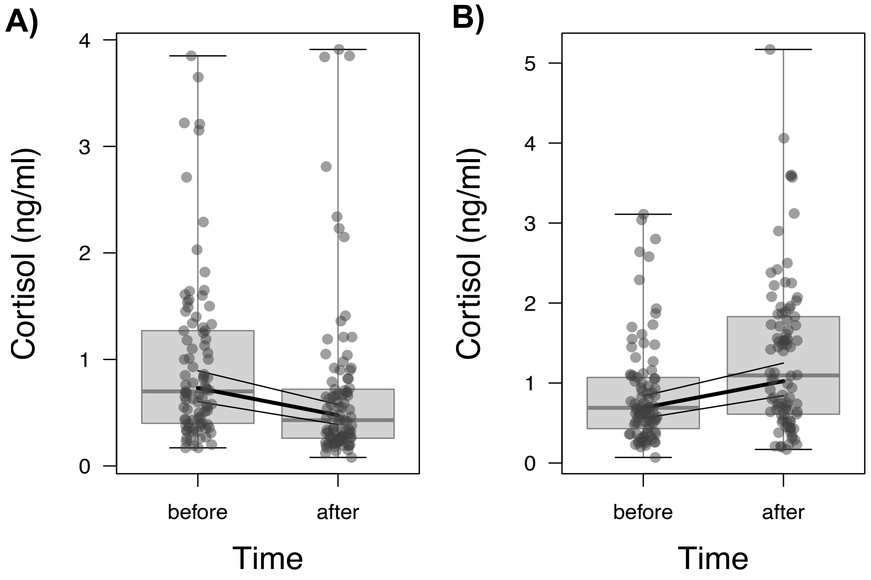


**Figure S9.** Cortisol (ng/ml) as a function of time point in **A)** food context and **B)** social context. Black points depict individual measurements. The bold black line depicts the fitted model and the outer lines the respective 95% confidence intervals.

## Supplementary methods - Handling missing values in PCA

### *Inhibition tests*

In order to be able to derive inhibition component scores from PCAs for all horses, even if one test was not completed, we ran four simple LMMs with the response variable that contained missing values as response variable. The first binomial LMM was set to have sessions to reach criterion in the RL test as response variable (based on 59 complete observations), while phase (factor: acquisition, reversal) was included as predictor and horse ID as random effect. The three missing values were imputed by using the *predict*-function (setting type to "response") and allowing for new levels of data. The extracted predicted values were used to replace the three missing values in the complete data set. Following the same procedure, we ran a second LMM with binomial error distribution (based on 61 complete observations) to impute the single

missing value for the number of correct choices in the RL test (i.e. 1st reversal session for horse that did not reach reversal phase) and again replaced the missing value in the complete data set. For the AB test (one horse did not reach B-phase), we ran two LMMs to impute the missing value for accuracy (binomial error distribution) and latency to cross the gap (poisson error distribution). For both models, trial (factor: A1-A3, B1-B5) was set as predictor and horse ID was included as random effect with trial as random slope. The model was based on 240 complete observations and the predicted values were used to replace the two missing values in the complete data set.

We calculated one PCA based on the complete observations (see Table 2 for variables that were used) and one PCA based on the full data set including the imputed values and compared them using a visualization of the strength of the factor loadings (Table S7). None of the loadings changed direction or changed the interpretation of the component (Inhibition-PCA: mean difference in loadings: 0.011 ± 0.097 (range: 0-0.194; see Table S7 and Coping-PCA: mean difference in loadings: 0.003 ± 0.034 (range: 0-0.189; see Table S8).

**Table S7.** Outcome of inhibition PCA based on A) complete observations and B) on data set including imputed values. Loadings are colour-coded with negative loadings in red, and positive loadings in green.

A) **PCA COMPLETE OBSERVATIONS**

| *Cumulative variance: 79.29 %* | | | | |
|---|---|---|---|---|
| | **Inhibition** | **Indecisiveness** | **Learning capacity** | **Flexibility** |
| **DG (max. delay)** | **0.530** | -0.227 | 0.024 | 0.117 |
| **DG (distract)** | **0.525** | -0.173 | 0.173 | -0.153 |
| **AB (ratio lat)** | **-0.489** | -0.226 | 0.080 | 0.281 |
| **AB (ratio acc)** | 0.232 | **0.569** | 0.020 | 0.047 |
| **RL (lat cor)** | 0.165 | **-0.466** | **-0.362** | -0.027 |
| **DG (FPT)** | 0.050 | **-0.451** | 0.037 | -0.303 |
| **RL (correct rev)** | 0.088 | 0.099 | **0.731** | -0.260 |
| **AB (A-trials)** | -0.056 | -0.313 | **0.526** | **0.539** |
| **RL (ratio session)** | 0.332 | 0.125 | -0.139 | **0.657** |
| **Eigenvalue** | 2.58 | 2.15 | 1.37 | 1.07 |
| **Variance** | 28.62 | 23.84 | 15.26 | 11.93 |

B) **PCA WITH IMPUTED VALUES**

| *Cumulative variance: 77.54 %* | | | | |
|---|---|---|---|---|
| | **Inhibition** | **Indecisiveness** | **Learning capacity** | **Flexibility** |
| **DG (max. delay)** | **0.536** | -0.218 | 0.107 | -0.010 |
| **DG (distract)** | **0.528** | -0.191 | 0.198 | -0.248 |
| **AB (ratio lat)** | **-0.474** | -0.222 | 0.042 | 0.095 |
| **AB (ratio acc)** | 0.259 | **0.532** | -0.127 | 0.116 |
| **RL (lat cor)** | 0.107 | **-0.530** | -0.226 | 0.082 |
| **DG (FPT)** | 0.048 | **-0.442** | 0.198 | -0.115 |
| **RL (correct rev)** | 0.076 | 0.282 | **0.707** | -0.066 |
| **AB (A-trials)** | -0.108 | -0.161 | **0.513** | **0.660** |
| **RL (ratio session)** | 0.332 | 0.041 | -0.281 | **0.675** |
| **Eigenvalue** | 2.45 | 2.17 | 1.30 | 1.07 |
| **Variance** | 27.17 | 24.06 | 14.44 | 11.87 |

Loadings > 0.35 are highlighted in bold as they were used for interpretation.

## *Coping Tests*

**Table S8.** Outcome of coping PCA based on a) original data set and b) data set including imputed variables. Loadings are colour-coded with negative loadings in red, and positive loadings in green.

A) **PCA COMPLETE OBSERVATIONS**

| *Cumulative variance: 66.82 %* | | | | | | |
|---|---|---|---|---|---|---|
| | **Nervousness** | **Stress** | **Anticipation** | **Reactivity** | **Oral motivation** | **Vigilance** |
| neigh | **0.372** | -0.180 | 0.056 | -0.151 | 0.084 | -0.063 |
| locomotion | **0.368** | 0.049 | 0.176 | -0.005 | 0.122 | 0.033 |
| defecate | **0.353** | -0.128 | 0.058 | 0.049 | 0.098 | -0.018 |
| shake (body + head) | 0.305 | 0.243 | -0.052 | -0.022 | 0.226 | 0.055 |
| snort | 0.297 | 0.251 | 0.143 | -0.023 | 0.060 | -0.208 |
| rear | 0.292 | 0.236 | 0.146 | 0.265 | -0.001 | -0.003 |
| head movements | 0.283 | 0.201 | -0.179 | 0.033 | 0.075 | 0.271 |
| agonistic | 0.025 | **0.382** | **-0.422** | 0.125 | -0.002 | 0.311 |
| baseline cortisol | -0.034 | **0.397** | 0.114 | **-0.526** | 0.161 | -0.109 |
| bar bite | -0.018 | **0.364** | -0.345 | 0.125 | -0.342 | -0.002 |
| oral behaviours | -0.106 | 0.306 | 0.142 | 0.328 | 0.304 | -0.155 |
| nicker | -0.029 | 0.048 | **0.511** | -0.063 | -0.061 | **0.489** |
| groom | -0.092 | -0.075 | -0.338 | -0.335 | 0.125 | 0.165 |
| check trough | -0.209 | 0.132 | 0.306 | 0.282 | -0.189 | **0.412** |
| cortisol difference | 0.228 | -0.229 | -0.114 | **0.430** | -0.238 | -0.222 |
| paw | 0.035 | 0.231 | 0.249 | -0.233 | **-0.574** | -0.289 |
| feed | -0.258 | 0.003 | 0.103 | 0.196 | **0.476** | -0.116 |
| alert | 0.263 | -0.266 | -0.040 | -0.111 | -0.072 | **0.400** |
| **Eigenvalue** | 4.88 | 2.40 | 1.52 | 1.16 | 1.07 | 1.00 |
| **Variance** | 27.08 | 13.33 | 8.44 | 6.44 | 5.97 | 5.56 |

B) **PCA WITH IMPUTED VALUES**

| *Cumulative variance: 66.93 %* | | | | | | |
|---|---|---|---|---|---|---|
| | **Nervousness** | **Stress** | **Anticipation** | **Reactivity** | **Oral motivation** | **Vigilance** |
| neigh | **0.369** | -0.184 | 0.051 | -0.132 | 0.058 | -0.073 |
| locomotion | **0.366** | 0.052 | 0.184 | -0.016 | 0.117 | 0.006 |
| defecate | **0.353** | -0.131 | 0.047 | 0.022 | 0.109 | -0.011 |
| shake (body + head) | 0.304 | 0.239 | -0.068 | -0.054 | 0.23 | 0.016 |
| snort | 0.296 | 0.254 | 0.145 | 0.025 | 0.026 | -0.239 |
| rear | 0.294 | 0.241 | 0.144 | 0.247 | 0.043 | 0.029 |
| head movements | 0.288 | 0.199 | -0.168 | -0.025 | 0.100 | 0.250 |
| agonistic | 0.018 | **0.385** | **-0.426** | 0.068 | 0.032 | 0.333 |
| baseline cortisol | -0.033 | **0.383** | 0.119 | **-0.546** | 0.093 | -0.150 |
| bar bite | -0.019 | **0.369** | -0.330 | 0.139 | -0.341 | 0.077 |
| oral behaviours | -0.111 | 0.309 | 0.126 | 0.286 | **0.355** | -0.128 |
| nicker | -0.027 | 0.034 | **0.507** | -0.155 | -0.017 | **0.445** |
| groom | -0.096 | -0.081 | -0.349 | **-0.352** | 0.083 | -0.024 |
| check trough | -0.209 | 0.131 | 0.306 | 0.258 | -0.124 | **0.453** |
| cortisol difference | 0.23 | -0.213 | -0.118 | **0.482** | -0.185 | -0.166 |
| paw | 0.036 | 0.235 | 0.261 | -0.100 | **-0.605** | -0.307 |
| feed | -0.261 | 0.008 | 0.099 | 0.176 | **0.476** | -0.143 |
| alert | 0.258 | -0.273 | -0.046 | -0.154 | -0.063 | **0.415** |
| **Eigenvalue** | 4.98 | 2.37 | 1.51 | 1.21 | 1.07 | 1.00 |
| **Variance** | 27.17 | 13.14 | 8.39 | 6.70 | 5.99 | 5.54 |

Loadings > 0.35 are highlighted in bold as they were used for interpretation.

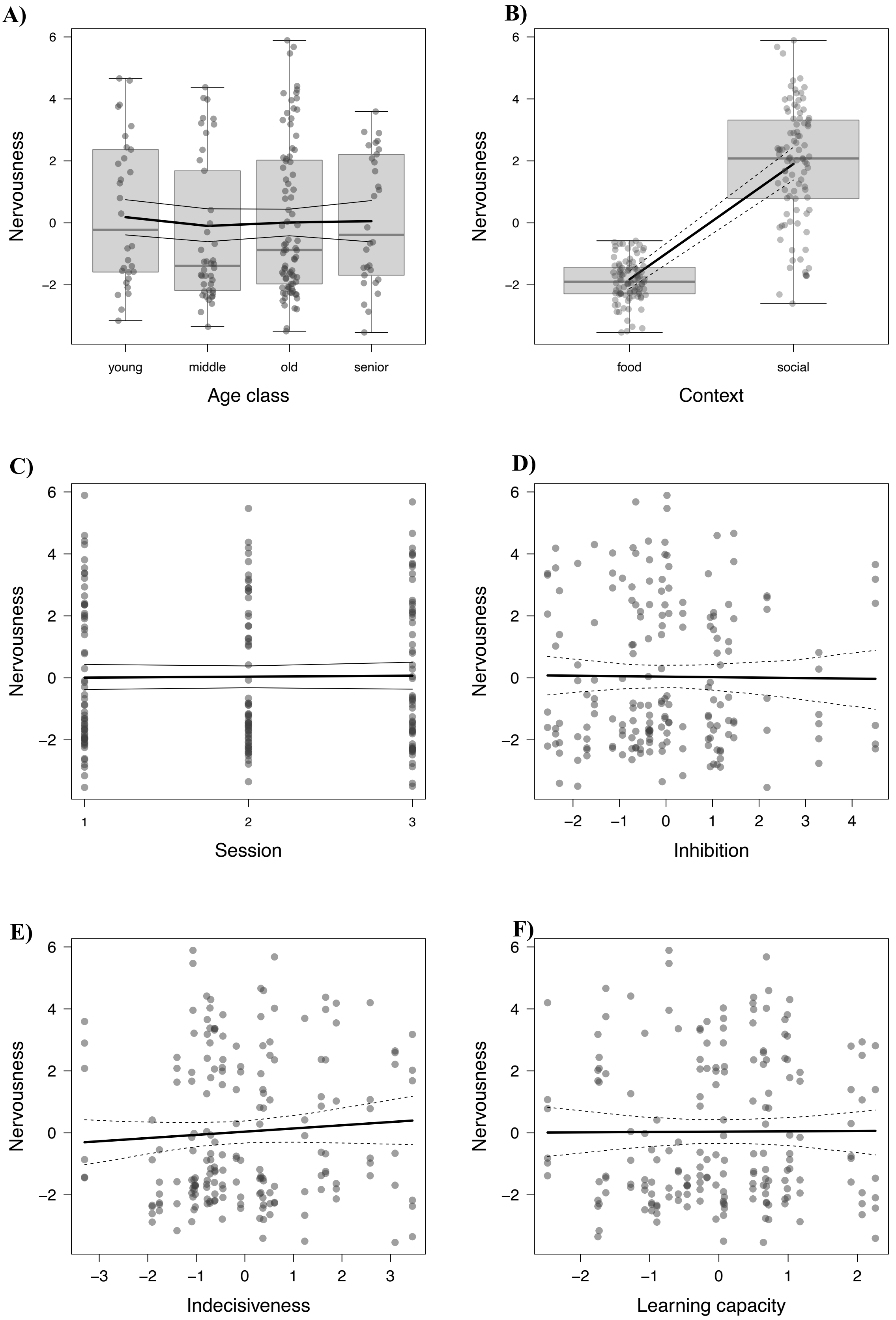
A)
B)
C)
D)
E)
F)
Nervousness
Age class
young
middle
old
senior
Context
food
social
Session
Inhibition
Indecisiveness
Learning capacity

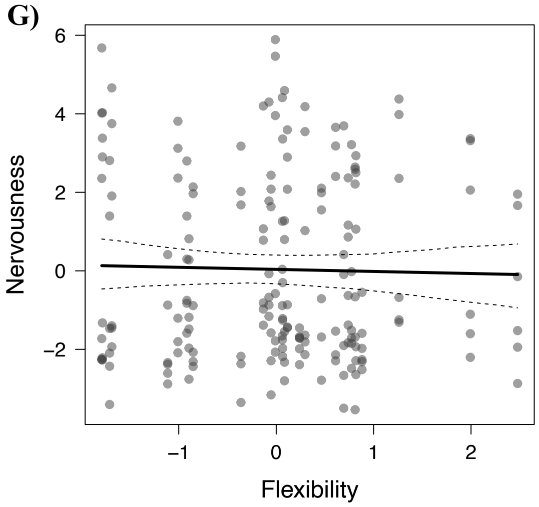


**Figure S10.** Effects of A) age class, B) context, C) session, D) inhibition component, E) indecisiveness component, F) learning capacity, and G) flexibility on *Nervousness* component. The thick black lines indicate the model estimates and the thinner lines, the 95% confidence intervals. The black points show individual data points.

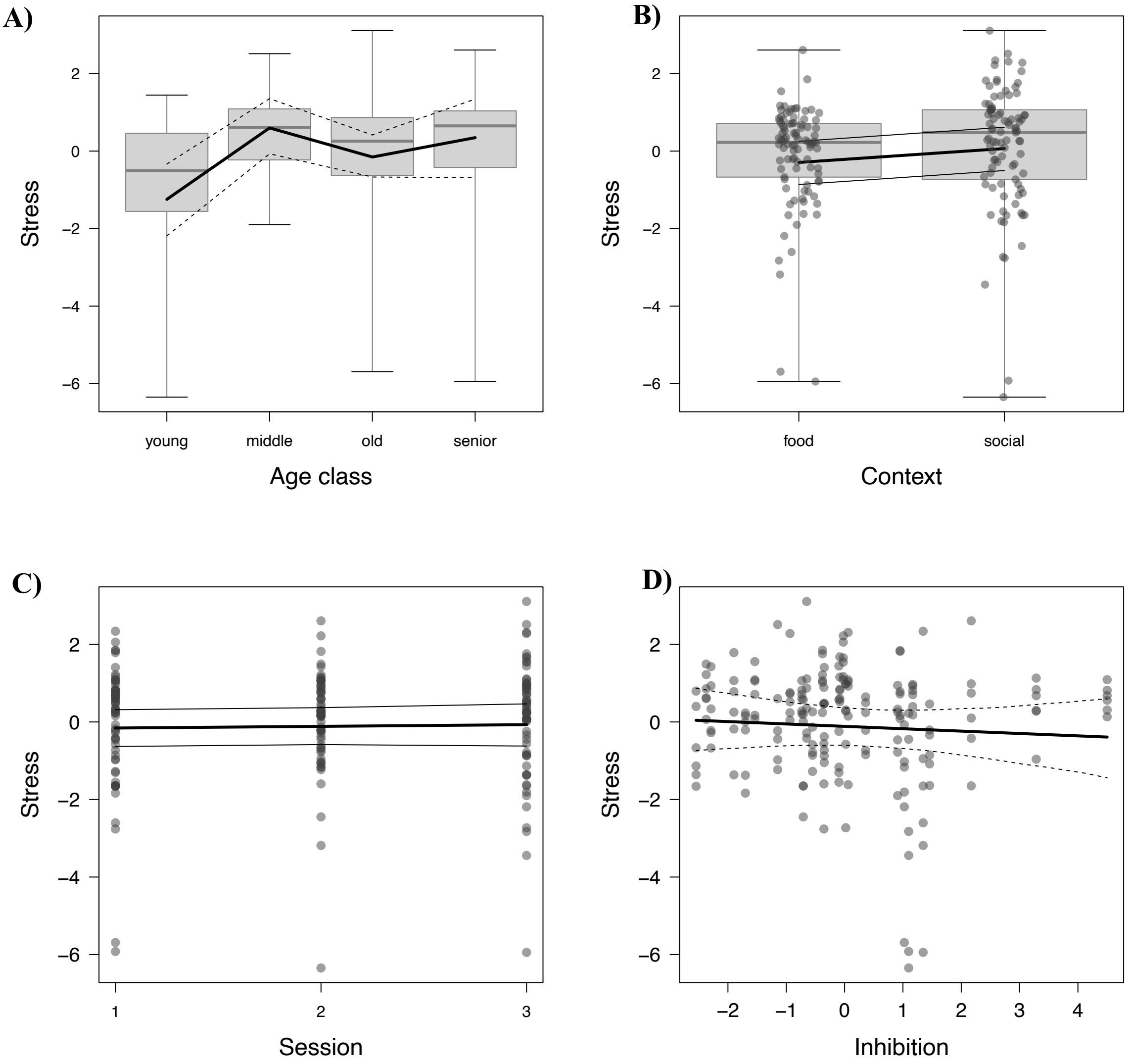

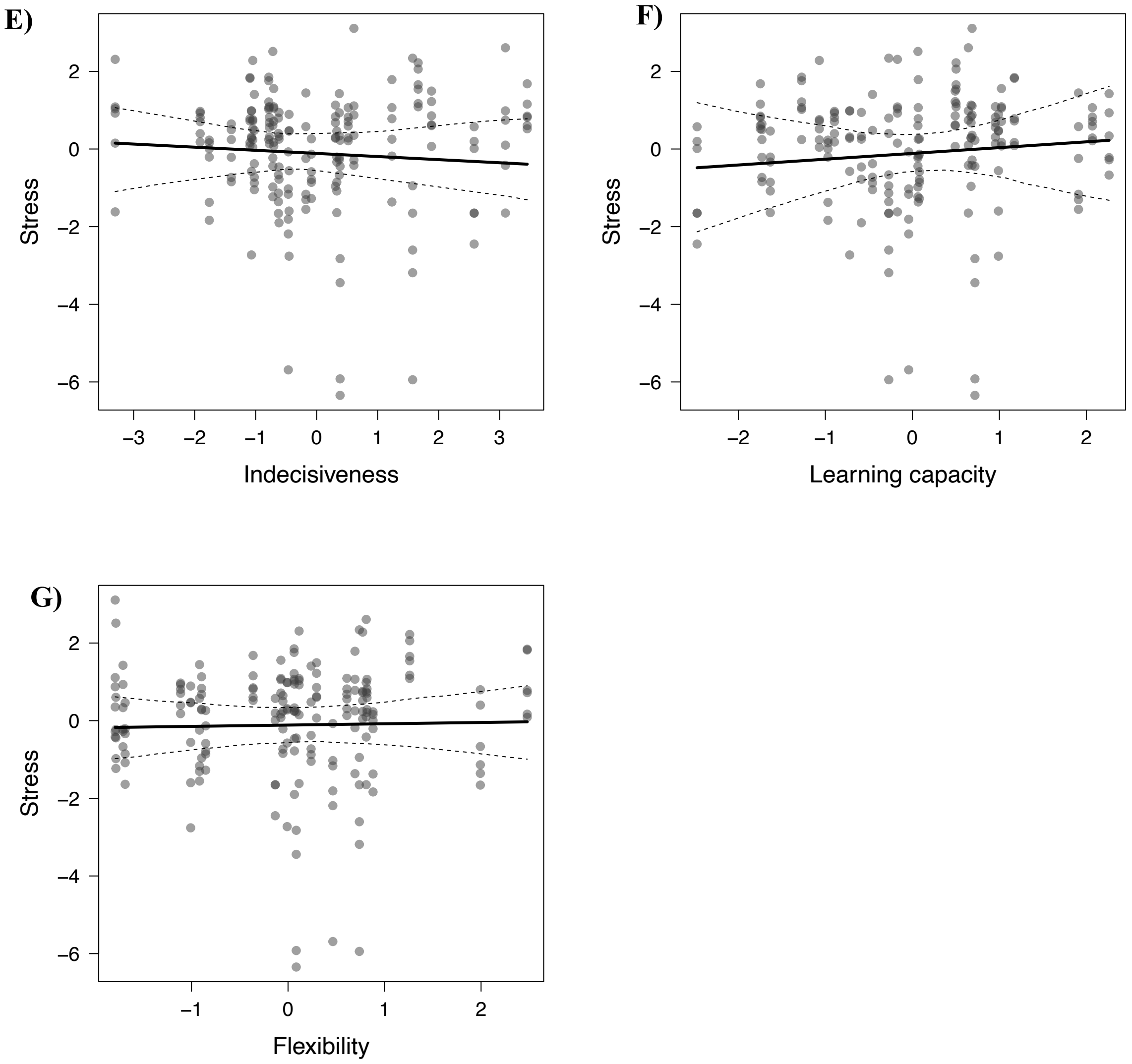


**Figure S11.** Effects of A) age class, B) context, C) session, D) inhibition component, E) indecisiveness component, F) learning capacity, and G) flexibility component on stress component. The thick black lines indicate the model estimates and the thinner lines, the 95% confidence intervals. The black points show individual data points.

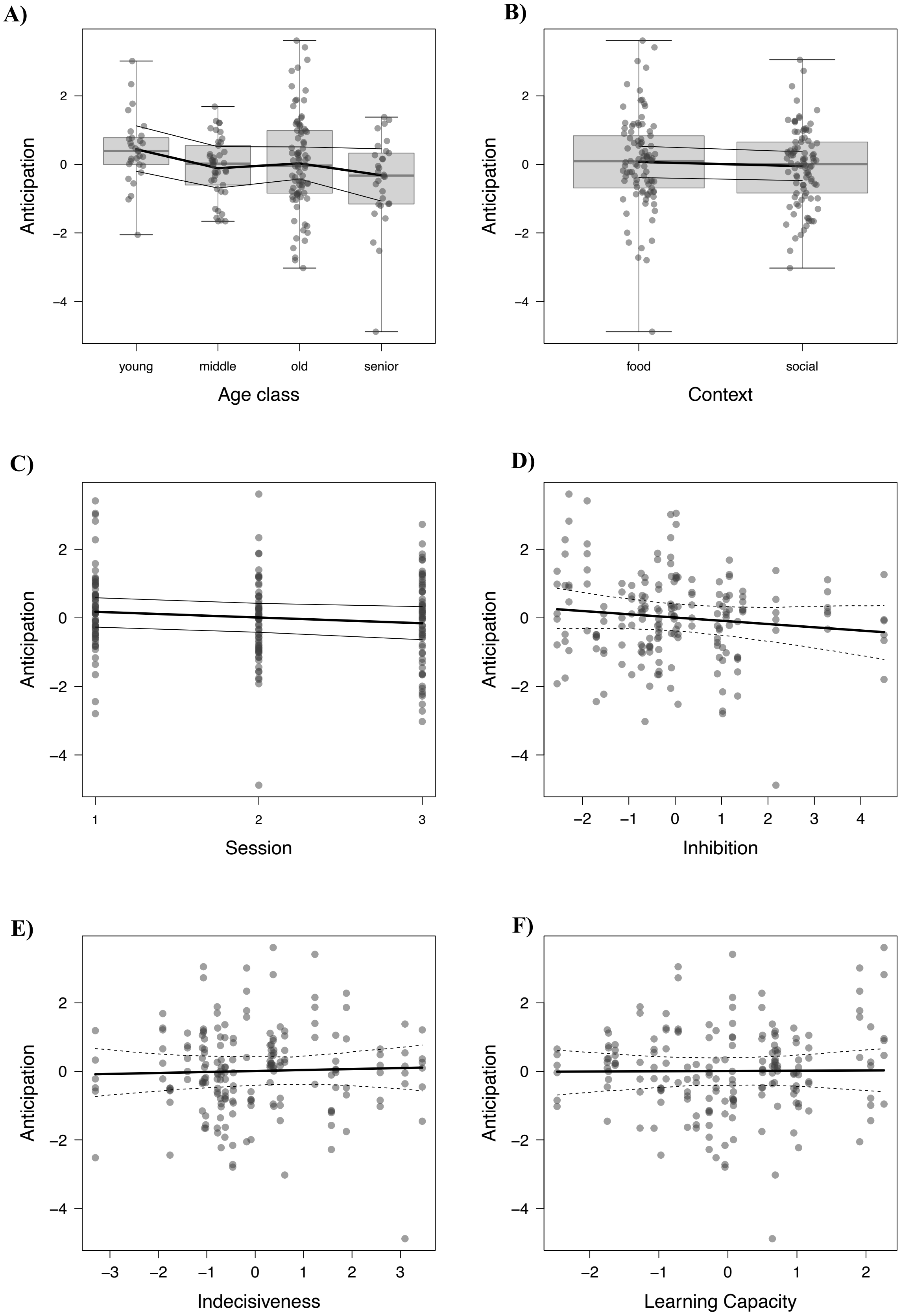
A)
B)
C)
D)
E)
F)
Anticipation
Age class
young
middle
old
senior
Context
food
social
Session
Inhibition
Indecisiveness
Learning Capacity

G)

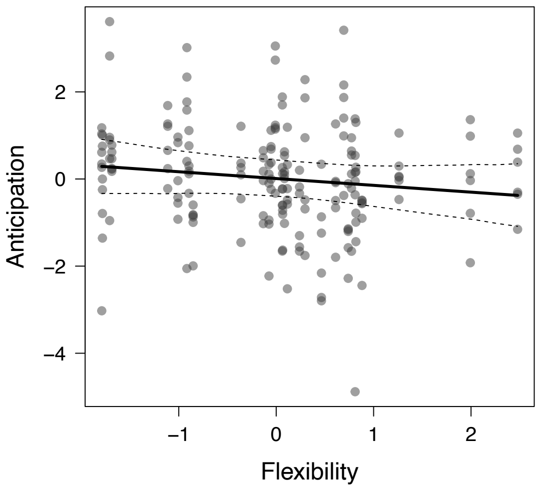


**Figure S12.** Effects of A) age class, B) context, C) session, D) inhibition component, E) indecisiveness component, F) learning capacity component, G) flexibility component on anticipation component. The thick black lines indicate the model estimates and the thinner lines, the 95% confidence intervals. The black points show individual data points.

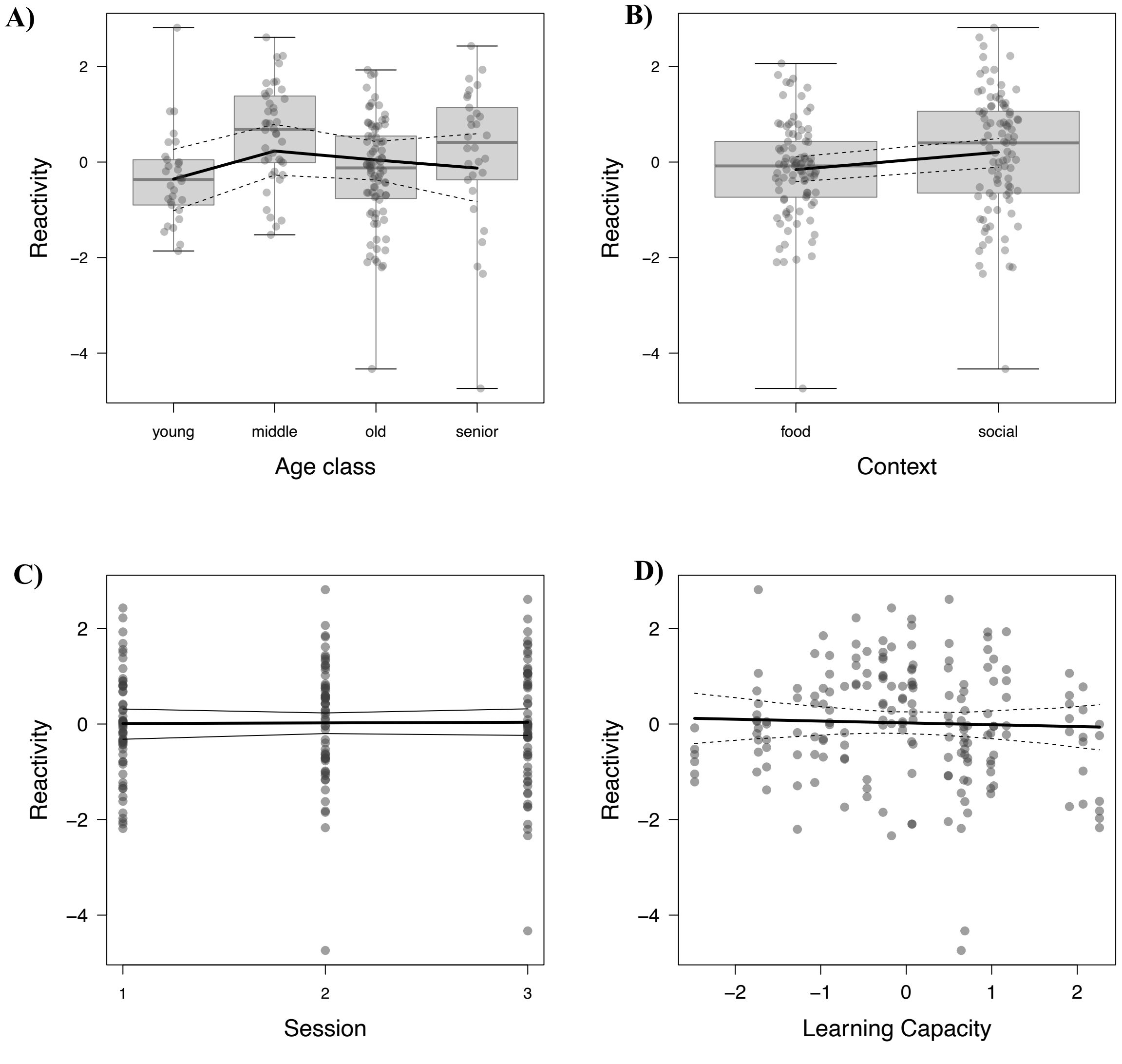

**Figure S13.** Effects of A) age class, B) context, C) session, and E) learning capacity component n reactivity component. The thick black lines indicate the model estimates and the thinner lines, the 95% confidence intervals. The black points show individual data points.

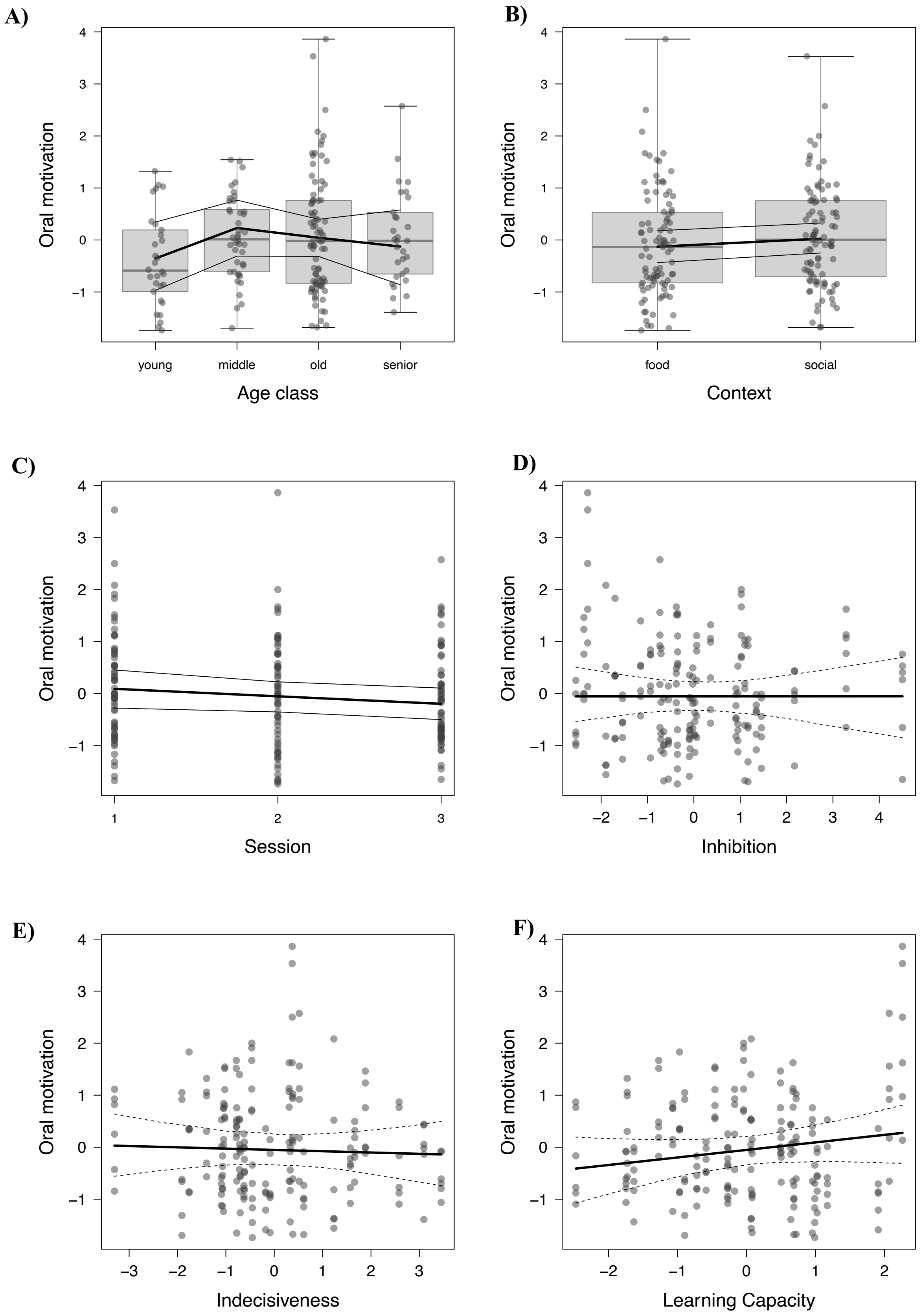

**G)**

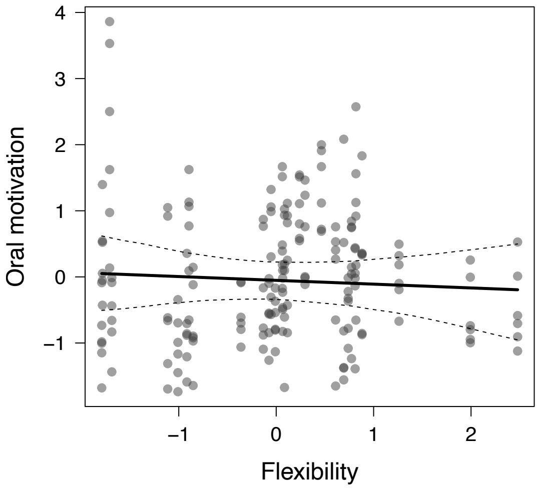


**Figure S14.** Effects of A) age class, B) context, C) session, D) inhibition component, E) indecisiveness component, F) learning capacity component, and G) flexibility component on calmness component. The thick black lines indicate the model estimates and the thinner lines, the 95% confidence intervals. The black points show individual data points.

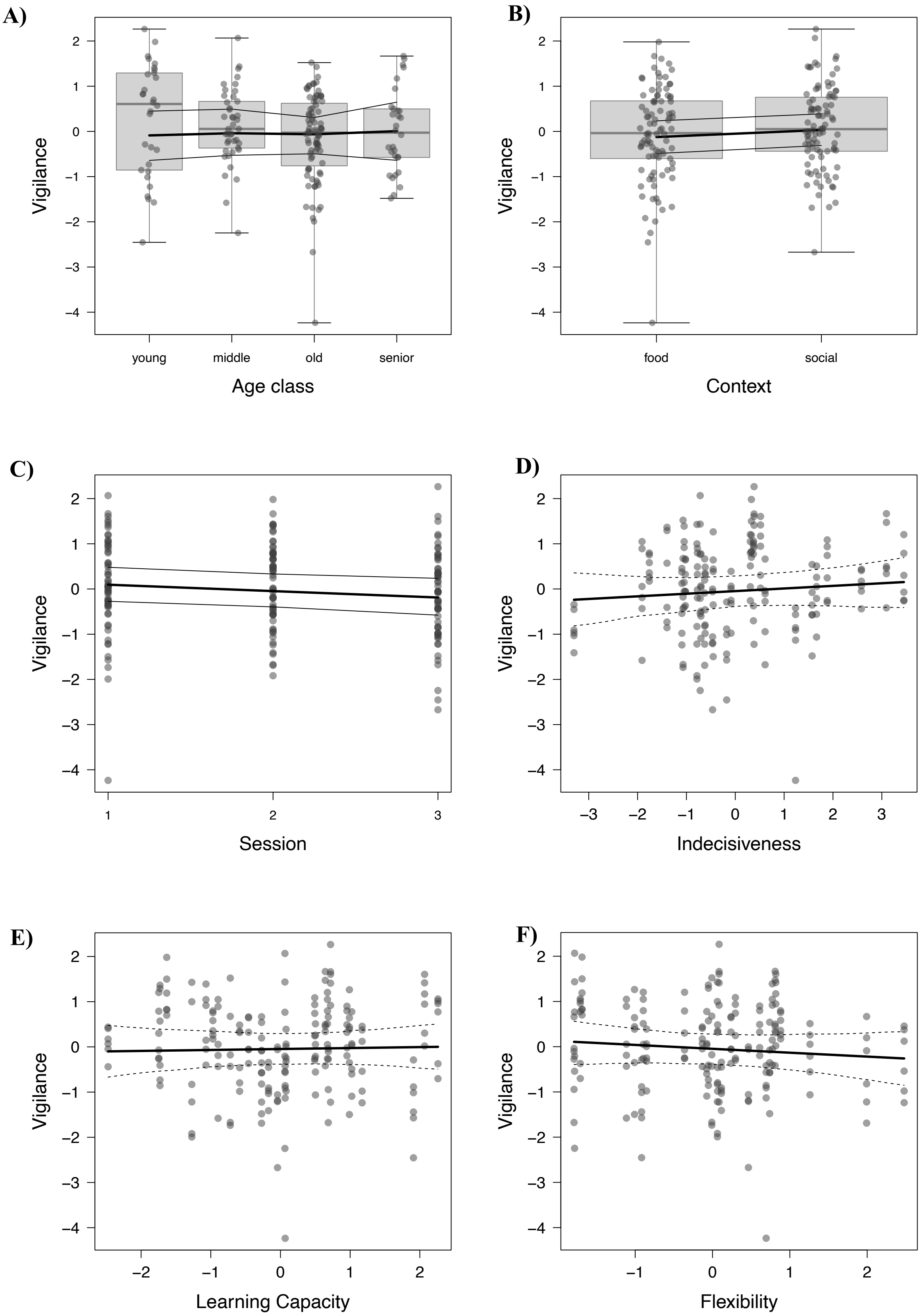


**Figure S15.** Effects of A) age class, B) context, C) session, D) indecisiveness component, E) learning capacity, and F) flexibility component on vigilance component. The thick black lines indicate the model estimates and the thinner lines, the 95% confidence intervals. The black points show individual data points.

## References


Aurich, J., Wulf, M., Ille, N., Erber, R., Von Lewinski, M., Palme, R., & Aurich, C. (2015). Effects of season, age, sex, and housing on salivary cortisol concentrations in horses. *Domestic Animal Endocrinology*, *52*, 11–16. https://doi.org/10.1016/j.domaniend.2015.01.003

Bohák, Zs., Szabó, F., Beckers, J.-F., Melo De Sousa, N., Kutasi, O., Nagy, K., & Szenci, O. (2013). Monitoring the circadian rhythm of serum and salivary cortisol concentrations in the horse. *Domestic Animal Endocrinology*, *45*(1), 38–42. https://doi.org/10.1016/j.domaniend.2013.04.001

Gamer, M., Lemon, J., Fellows, I., & Singh, P. (2019). *Various Coefficients of Interrater Reliability and Agreement* [Computer software]. https://cran.r-project.org/package=irr

Massányi, M., Halo, M., Mlyneková, E., Kováčiková, E., Tokárová, K., Greń, A., Massányi, P., & Halo, M. (2023). The effect of training load stress on salivary cortisol concentrations, health parameters and hematological parameters in horses. *Heliyon*, *9*(8), e19037. https://doi.org/10.1016/j.heliyon.2023.e19037

Schmidt, A., Möstl, E., Wehnert, C., Aurich, J., Müller, J., & Aurich, C. (2010). Cortisol release and heart rate variability in horses during road transport. *Hormones and Behavior*, *57*(2), 209–215. https://doi.org/10.1016/j.yhbeh.2009.11.003
